# Data-driven and mechanistic modelling of PLGA nanoparticle formation in hydrodynamic flow focusing microfluidics

**DOI:** 10.1039/d6lc00612d

**Published:** 2026-09-21

**Authors:** Muhammad Mubashar Saeed, Muhammad Turab, Lasse H. E. Thamdrup, Anja Boisen, Eadaoin Carthy, Nicholas Dunne, David Kinahan

**Affiliations:** a ML-Labs Centre for Research Training, Dublin City University Dublin Ireland; b School of Mechanical and Manufacturing Engineering, Dublin City University Dublin Ireland Muhammad.saeed3@mail.dcu.ie; c Biodesign Europe, Dublin City University Dublin Ireland; d RAPID Institute, Dublin City University Dublin Ireland; e School of Computing, Dublin City University Dublin Ireland; f IDUN Section, Department of Health Technology, Technical University of Denmark Kongens Lyngby Denmark; g Centre for Medical Engineering Research, Dublin City University Dublin Ireland; h Advanced Manufacturing Research Centre (I-Form), School of Mechanical and Manufacturing Engineering, Dublin City University Dublin Ireland; i School of Pharmacy, Queen's University Belfast Belfast UK; j Advanced Processing Technology Research Centre, Dublin City University Dublin Ireland; k Research Centre for Medical Devices (CÚRAM), Biomedical Sciences, University of Galway Galway Ireland

## Abstract

Microfluidic devices offer precise control over polymeric nanoparticle (NP) synthesis. However, rational design of process optimization to obtain a target NP size remains challenging because the relationship between operating parameters, mixing dynamics, and particle formation is nonlinear. Here, we integrate mechanistic analysis, statistical design of experiments (DoE), and machine learning (ML) to develop predictive and inverse design frameworks for the synthesis of poly(lactic-*co*-glycolic acid) (PLGA) NPs. The effects of flow rate ratio (FRR), PLGA concentration, and microchannel width were systematically investigated using hydrodynamic flow focusing (HFF) microfluidic devices. Mixing time analysis demonstrated that NP size is primarily governed by solvent-to-antisolvent mixing, with equivalent mixing times producing comparable particle sizes despite different combinations of FRR and channel width. A Box–Behnken DoE yielded a highly predictive quadratic model (*R*^2^ = 0.9979), identifying microchannel width as the dominant design variable, followed by PLGA concentration and FRR. Eight machine learning algorithms were subsequently developed and evaluated with gradient boosting achieving the highest predictive accuracy (test *R*^2^ = 0.9900, RMSE = 2.65 nm). Finally, inverse design experiments were performed for target NP sizes of 80, 120, and 160 nm using DoE and a selected subset of ML models. Process conditions predicted by the developed models were implemented experimentally. The gradient boosting model produced NP sizes in closest agreement with the preassigned targets, outperforming DoE and other ML models. This combined mechanistic and data-driven framework enables reliable prediction and inverse design of PLGA NP synthesis and provides a practical strategy for intelligent and on-demand microfluidic-based synthesis of polymeric nanomedicines.

## Abstract 2

Microfluidic devices offer precise control over polymeric nanoparticle (NP) synthesis.

## Graphical abstract

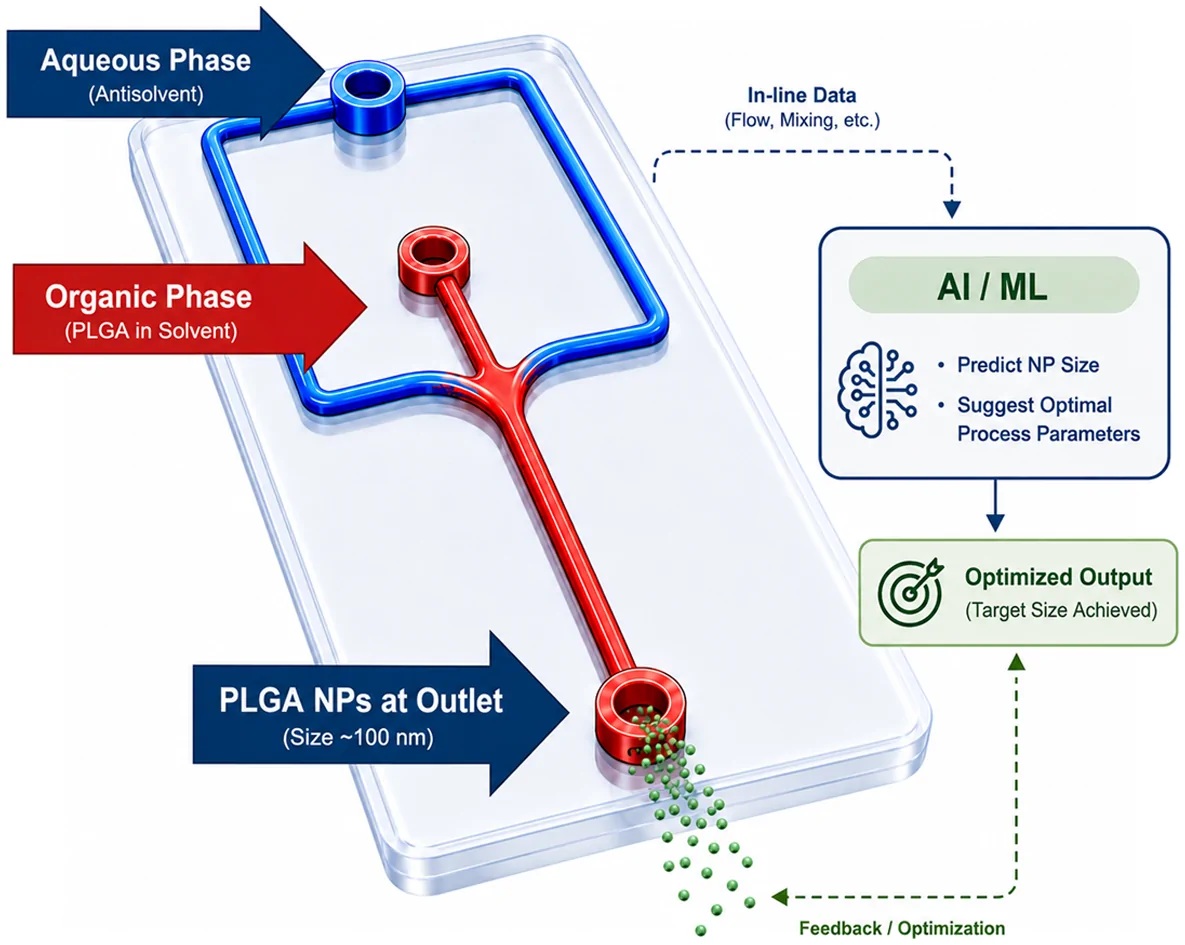

## 1. Introduction

Polymeric nanoparticles (NPs) based on poly(lactic-*co*-glycolic acid) (PLGA) are among the most widely studied colloidal carriers in nanomedicine due to their biodegradability, biocompatibility, and established clinical relevance.^1,2^ Their therapeutic performance is strongly dependent on physicochemical properties, particularly particle size, polydispersity index (PDI), and colloidal stability. Particle size influences key biological processes including circulation time, biodistribution, cellular uptake, and drug release kinetics, while a low PDI is essential for maintaining a uniform and reproducible NP population. Therefore, precise control over NP size is critical for achieving predictable therapeutic outcomes.^3–6^

Despite this importance, NP size determination remains a post-synthesis characterization step as it can be measured only after synthesis is complete, typically *via* dynamic light scattering (DLS) or, *e.g.* scanning or transmission electron microscopy, with limited ability to anticipate the resulting particle size before synthesis. As a result, researchers are largely confined to iterative, trial-and-error adjustment of synthesis parameters, repeating batches until a target size is obtained through empirical experience rather than by design. Most studies remain limited to parametric investigations that examine how individual process variables influence NP attributes without establishing a predictive link between synthesis conditions and the resulting NP size.^7–9^

The inability to predict NP size directly from synthesis conditions increases experimental effort, material consumption, and development time. Consequently, predictive models capable of estimating NP size prior to synthesis are increasingly recognized as an essential step toward rational process design and efficient NP manufacturing.

Conventional bulk nanoprecipitation methods offer limited and largely empirical control over process parameters. Mixing is governed by uncontrolled turbulent or diffusive processes influenced by different factors such as stirring rate and operator technique. which make predictable tuning of particle size difficult and results in poor batch-to-batch reproducibility.^10^ Microfluidic synthesis using hydrodynamic flow focusing (HFF) addresses this limitation directly by confining nanoprecipitation to a well-defined laminar flow field. HFF transforms particle formation from being unpredictable and operator-dependent into a deterministic process governed by externally controllable parameters such as channel geometry, flow rate ratio (FRR), and solute concentration.^11^ These controllable inputs regulate mixing time and solvent exchange, providing an opportunity to establish quantitative relationships between synthesis conditions and NP size.

Recent studies on microfluidic synthesis of PLGA NPs have primarily focused on the development of complex device architectures and mixing enhancement strategies, including herringbone,^12,13^ Tesla,^14^ and other passive micromixer designs.^11^ Parallelised architectures have also been employed to produce NPs across multiple sizes within a single chip. Devices incorporating 128 mixing channels^15^ or up to 256 silicon channels^16^ preserve particle quality while increasing throughput by two orders of magnitude. In parallel, active approaches also span the micro- and nanoscale by employing different active methods to intensify solvent-to-antisolvent exchange for PLGA nanoprecipitation.^11,17,18^

While these approaches have significantly improved NP synthesis, comparatively little attention has been given to developing predictive frameworks capable of quantitatively linking microfluidic process parameters to NPs attributes. As a result, NP formation in microfluidic systems remains largely empirical, despite the highly controllable and predictable nature of microscale transport phenomena.

Design of experiments (DoE) provides a statistically robust approach for identifying the individual and combined effects of varying process variables while minimizing experimental effort.^19,20^ Machine learning (ML) has recently emerged as an effective tool for the design and optimization of microfluidic devices by reducing the need for extensive experimental studies and computationally expensive CFD simulations. Equivalent hydraulic electrical circuit models and CFD generated datasets are widely used to train ML algorithms for predicting flow behaviour, mixing efficiency, and particle characteristics. Deep learning techniques, including artificial neural networks (ANNs) and physics-informed neural networks (PINNs), have further improved the accuracy of device performance prediction and inverse design optimization. This integration of ML with microfluidics is expected to accelerate the development of intelligent, high-throughput microfluidic platforms for NP synthesis.^21–24^ Mihandoost *et al.* developed ML models to predict the size of PLGA NPs using a literature-derived dataset.^25^ Similarly, Hanari *et al.* applied ML algorithms to predict the encapsulation efficiency and drug loading of PLGA NPs synthesized using microfluidic platforms.^26^ While some recent studies have demonstrated the potential of ML for NP process optimization, most models treat operating parameters as black box inputs without explaining the underlying transport phenomena governing NP formation.^25,27,28^ In particular, the mechanistic role of mixing time, which directly influences solvent exchange, supersaturation development, and NP nucleation and growth, is often overlooked. Furthermore, few studies have systematically investigated the combined influence of channel geometry, FRR and polymer concentration within a unified predictive framework.

To address these limitations, we employed a simple HFF-based microfluidic platform with straight channels rather than complex micromixer architectures. This simplified configuration was intentionally selected to isolate and investigate the fundamental effects of microchannel width, FRR, and PLGA concentration on mixing behaviour and NP formation. Mixing times associated with different synthesis conditions were also quantified and correlated with experimentally measured NP sizes, providing a mechanistic basis for understanding the relationship between microfluidic transport phenomena and particle formation.

Building on the experimental study and mixing time analysis, DOE was employed to quantify the individual and combine effects of channel width, FRR, and PLGA concentration on NP size, while ML models were trained to predict particle size directly from synthesis conditions. The trained model was subsequently used to identify operating conditions required to achieve user-defined target sizes.

Unlike conventional trial-and-error optimization approaches, which require repeated experimental adjustments without establishing quantitative relationships between synthesis conditions and NP properties, the proposed framework integrates mechanistic analysis, statistical design, and machine learning-based prediction to reduce experimental efforts, improve process understanding, and enable rational process optimization. Mixing time analysis provides mechanistic insight into how microfluidic process parameters regulate NP formation and size, while DoE identifies the contribution and interactions of critical process parameters and ML enables prediction of NP size under defined synthesis conditions. Together, these complementary approaches establish a predictive framework for the rational design and optimization of microfluidic assisted PLGA NP synthesis. Furthermore, this framework provides a transferable strategy that can be extended beyond PLGA NPs by incorporating additional formulation variables, alternative polymer systems, different microfluidic device architectures, and other NP quality attributes, supporting the development of more generalizable and intelligent microfluidic manufacturing platforms.

## 2. Materials and methods

A SYLGARD™ 184 Silicone Elastomer kit was obtained from Dow Chemical Company (Stade, Germany). PMMA foils were purchased from König Deutschland GmbH (Schorndorf, Germany). 4-inch silicon substrates were purchased from Siegert Wafer GmbH (Aachen, Germany). Poly(d,l-lactide-*co*-glycolide) (PLGA; lactide : glycolide 50 : 50, *M*_w_ 38 000–54 000), poly(vinyl alcohol) (PVA; *M*_w_ 9000–10 000), and acetonitrile (ACN) were purchased from Merck Sigma-Aldrich (Germany).

### 2.1 Microfluidic chip fabrication

As described in a previous publication,^9^ microfluidic devices were made by polydimethylsiloxane (PDMS) casting on pre-made 250 μm thick polymethyl methacrylate (PMMA) replicas of silicon masters made by conventional top-down planar processing. In the context of the current work, silicon masters featuring microfluidic channels with widths of 100, 200 and 300 μm were fabricated. The channel depth was kept constant at 100 μm ± 2 μm and an overview of the overall design of the fluidic chips can be found in Fig. 1b. Single side polished 100 mm diameter, silicon wafers with a thickness of 525 μm were used from stock. Initially, a 2 μm thick layer of AZ MiR 701 (MicroChemicals GmbH, Ulm Germany) was spin-coated and maskless UV lithography (MLA150 WM I, Maskless Aligner, Heidelberg Instruments, Heidelberg, Germany) was employed for defining the photoresist etch masks. To obtain microfluidic channels with vertical sidewalls and a depth of 100 μm, anisotropic inductively coupled deep reactive ion etching (STS Pegasus, SPTS Technologies, Newport, UK) was used. After the dry etch, the remaining photoresist was stripped using oxygen plasma ashing in a plasma asher (PVA TePla Resist Ashing System GIGAbatch 380M, PVA TePla America LLC, Corona, CA, USA). Finally, the etched masters were immersed in a dedicated cleaning solution comprising concentrated H_2_SO_4_ with (NH_4_)_2_S_2_O_8_ salt added just prior to immersion. The temperature of the solution was kept at 80 °C which ensures complete removal of organic traces. Hot embossing was used for making PMMA replicas featuring protrusions corresponding to the microfluidic network. Before hot embossing, the clean silicon masters were subjected to molecular vapor deposition (MVD 100 Molecular Vapor Deposition System, Applied Microstructures Inc., San Jose, CA, USA) where a self-assembled conformal monolayer of 1*H*,1*H*,2*H*,2*H*-perflourodecyltrichlorosilane (FDTS) was deposited. The covalently bound FDTS functions as an effective anti-stick layer exhibiting very low surface free energy. This effectively ensures a smooth replication cycle during hot embossing which was conducted on a Compact Nanoimprint Tool (CNI v2.0, NIL Technology ApS, Lyngby, Denmark). Circular PMMA foils having a diameter of approximately 95 mm were pre-cut and hot embossing was conducted using an imprint chamber vacuum of 10–70 mbar, an imprint temperature of *T* = 170 °C, a pneumatic embossing pressure of 6 bar and an embossing time of 30 min. Hot embossing was terminated at 50–60 °C and the PMMA replications were demolded from the silicon master by hand. This resulted in full replication of all topographies on the silicon masters. The PMMA replicas were then used for making PDMS copies by casting.

**Fig. 1:**
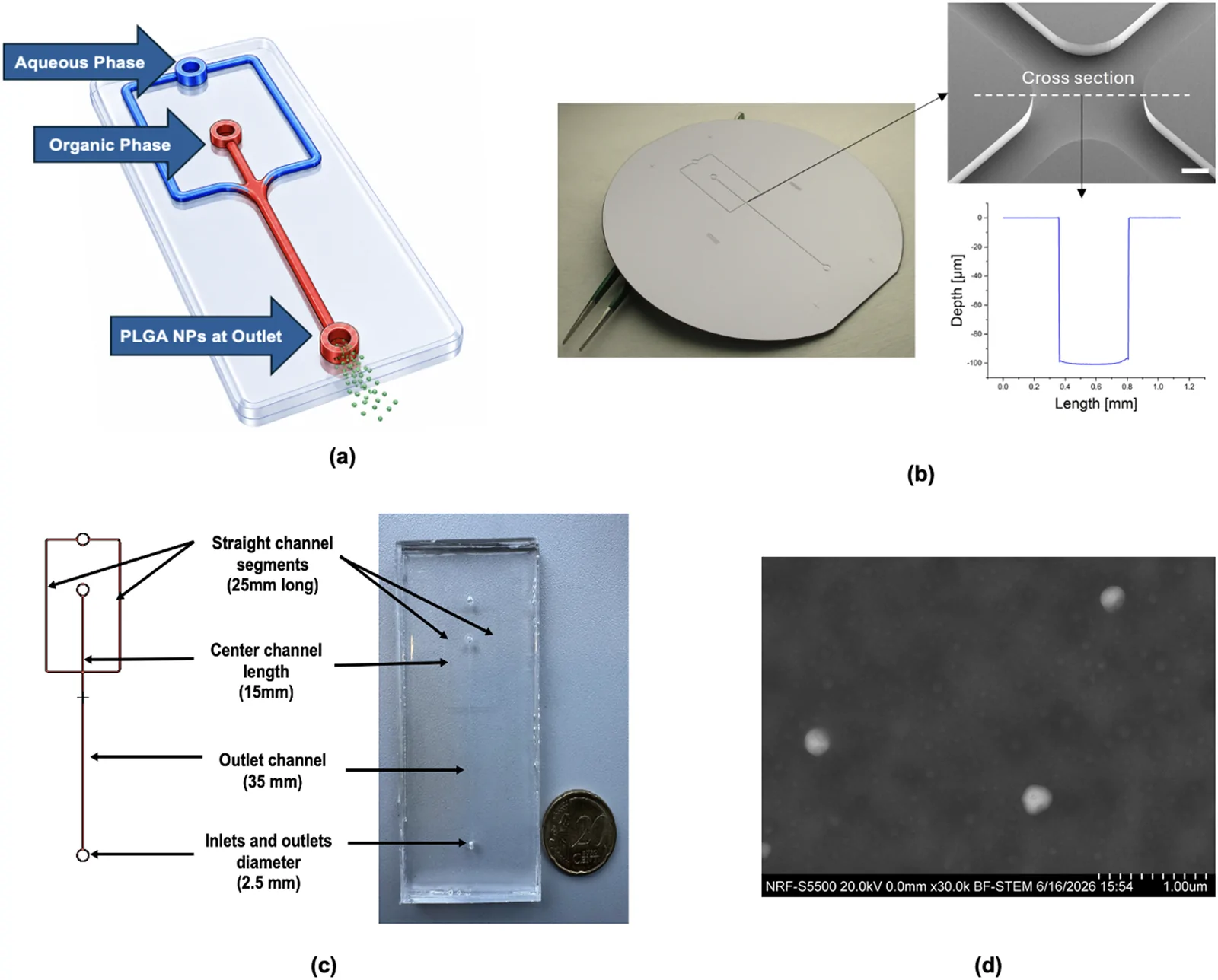
Hydrodynamic flow focusing based microfluidic setup for PLGA NP synthesis. (a) Schematic illustration of the HFF process used for PLGA NP synthesis, where a PLGA solution in acetonitrile (organic phase) is injected through the central inlet and focused by two aqueous streams, and the resulting NP suspension is collected at the outlet. (b) Fabricated Si master with SEM image showing the fluidic channel intersection and cross-sectional profile obtained from optical profilometry confirming a channel depth of 100 μm. (c) Photograph and dimensional layout of the fabricated PDMS microfluidic device used for PLGA NP synthesis. (d) SEM image of the synthesized PLGA NPs.

For fabrication of the PDMS microfluidic chips, different PDMS base to curing agent ratios were used for the two layers: a 5 : 1 (w/w) ratio was applied for the bottom layer containing the microchannels, while a 15 : 1 (w/w) ratio was used for the top cover layer. This differentiated formulation was designed to balance mechanical stability and bonding performance. The bottom layer was prepared with a higher curing agent content (5 : 1) to achieve a higher crosslinking density, providing sufficient mechanical rigidity and maintaining channel integrity during operation. In contrast, the top layer was fabricated using a 15 : 1 composition to enhance interfacial bonding through increased polymer chain mobility and interdiffusion during oxygen plasma treatment.^9,29^ Both PDMS mixtures were degassed under vacuum and cured overnight at 70 °C. After thermal curing, the layers were carefully peeled from the moulds, and inlet and outlet holes were created in the bottom layer using a punch. The layers were cleaned to remove dust and debris before oxygen plasma activation at 75% power for 1 min. The activated surfaces were aligned and bonded under compressive force at 70 °C for 20 min, resulting in robust and leak-free sealing. The completed PDMS microfluidic chips were subsequently used for NP synthesis experiments (Fig. 1a), and the device dimensions and photograph are shown in Fig. 1c.

### 2.2 PLGA nanoparticle synthesis

PLGA NPs were synthesized using a hydrodynamic flow focusing (HFF) microfluidic device, in which a central organic stream was focused by two co-flowing aqueous streams. As shown in Fig. 1a, the microfluidic device consisted of two inlets and one outlet. One inlet was used for the aqueous phase and bifurcated into two side channels leading to the flow-focusing junction, while the second inlet was used for the organic phase.

An aqueous solution of PVA (0.25% w/v) was delivered through the aqueous inlet using KF Technology syringe pumps, and the organic phase comprising PLGA dissolved in acetonitrile was introduced through the central inlet. Upon convergence at the flow-focusing intersection, rapid solvent exchange between the organic and aqueous phases induced nanoprecipitation of PLGA, resulting in the formation of NPs.

The influence of synthesis parameters on NP formation was investigated by varying the FRR, defined as the ratio of the organic phase inlet flow rate (*Q*_c_) to the aqueous phase inlet flow rate (*Q*_s_) together with the PLGA concentration and microchannel width. The total flow rate (TFR) was kept constant at 110 μL min^−1^ throughout all the experiments to ensure comparable mixing conditions. The resulting PLGA NP suspensions were continuously collected from the outlet and subsequently characterized.

### 2.3 PLGA NP characterization

#### 2.3.1 Dynamic light scattering analysis

The hydrodynamic diameter and polydispersity index (PDI) of the PLGA NPs were determined at 25 °C using a Malvern Zetasizer Ultra system (Malvern Panalytical, UK). Prior to analysis, 100 μL of NP suspension was diluted with 900 μL of deionized (DI) water and transferred into DTS0012 disposable polystyrene cuvettes (10 mm × 10 mm). Samples were equilibrated at the measurement temperature for 30 s before data acquisition. For each sample, three independent measurements were performed to evaluate measurement reproducibility, and the average values were reported.

The colloidal stability of the NP suspensions was assessed by repeating dynamic light scattering (DLS) measurements after 24 hours, 72 hours, one week and one month of storage. No significant changes in hydrodynamic diameter or PDI were observed over this period, indicating good colloidal stability of the PLGA NPs.

#### 2.3.2 Scanning electron microscopy

The morphology of the PLGA nanoparticles was examined using a field-emission scanning electron microscope (FESEM; Hitachi S-5500). NP suspensions were prepared by depositing a droplet onto 300-mesh copper grids (Agar Scientific) and allowing the samples to dry under ambient conditions. The dried samples were subsequently imaged at accelerating voltages and beam currents of 10–20 μA to evaluate particle morphology and size distribution (Fig. 1d).

### 2.4 Design of experiments analysis

Design of experiments (DoE) was performed using Design-Expert® software (Stat-Ease Inc., Minneapolis, MN, USA) to investigate and optimize the microfluidic synthesis parameters affecting PLGA NP formation. A three-factor, three-level Box–Behnken design (BBD) was employed, with FRR range (0.05–0.15), PLGA concentration (2.5–7.5 mg mL^−1^), and microchannel width (*w* = 100–300 μm) were selected as independent variables. The hydrodynamic diameter of the NPs was used as the response variable. A total of 17 experimental runs were generated and conducted in randomized order to minimize systematic bias.

Response surface methodology (RSM) was used to evaluate the effects of the selected process variables on NP size. Experimental data were fitted to a second-order polynomial model, and analysis of variance (ANOVA) was performed to assess model significance and determine the individual and interactive effects of the investigated factors. Model adequacy was evaluated based on the coefficient of determination (*R*^2^), adjusted *R*^2^, and lack-of-fit test.

### 2.5 Machine learning predictive models

#### 2.5.1 Dataset description

The dataset consists of 81 experiments of four variables including FRR, PLGA concentration, channel width, and NP size. The first three variables were used as predictors, while NP size was used as the continuous target variable. Each predictor had three levels: FRR = 0.05, 0.10, and 0.15; PLGA concentration = 2.5, 5.0, and 7.5 mg ml^−1^; and width = 100, 200, and 300 μm. In order to study the impact of FRR exclusively, the total flow rate (TFR: *Q*_c_ + *Q*_s_) was kept constant at 110 μL min^−1^. The design therefore contained 27 unique experimental combinations, with three repetitions per combination. No missing values were present in the dataset. Descriptive analysis showed that the mean target NP size increased with increasing FRR, PLGA concentration, and width.

#### 2.5.2 Data preprocessing and train–test split

The input matrix was defined as *X* = [FRR, PLGA concentration, channel width] and the response variable was defined as: *y* = NP size. The dataset was divided into training and testing subsets using an 80 : 20 split, producing 64 training samples and 17 testing samples. The split was performed using the train–test split function, which randomly partitions arrays into train and test subsets. For models sensitive to feature scale, namely linear regression, ridge regression, lasso regression, polynomial regression, and support vector regression, the predictors were standardized using StandardScaler. This transformation removes the mean and scales features to unit variance, calculated from the training samples and then applied to the test samples.

#### 2.5.3 Predictive modelling

Eight regression models were applied using the scikit learn library (Python) to predict NP size from three process parameters: FRR, PLGA concentration, and microfluidic channel width (*w*). Ordinary least-squares linear regression served as the baseline. Ridge regression (*α* = 1.0) and lasso regression (*α* = 0.1) were included as regularised alternatives, applying L2 and L1 penalties, respectively; the latter also provides implicit feature selection by shrinking coefficients toward zero. To capture nonlinear and interaction effects, second-degree polynomial features were generated prior to fitting a linear model.

Three ensemble and tree-based approaches were also evaluated. A decision tree regressor (max_depth = 5) was trained to partition the feature space according to classification and regression trees (CART) principles. Random forest regression (100 trees) extended this by averaging predictions across trees trained on bootstrapped subsamples, reducing overfitting. Gradient boosting regression (100 estimators) employed sequential additive learning, with each weak learner correcting the residuals of the prior ensemble. Finally, support vector regression was implemented with a radial basis function kernel (*C* = 100, *γ* = 0.1), enabling the modelling of nonlinear relationships within a specified error margin. These specific hyperparameter values were selected based on standard default settings for these algorithms, as the study focused on comparing the performance of different model architectures rather than exhaustive hyperparameter optimization.

#### 2.5.4 Inverse prediction modelling

To enable process parameter estimation for a target NP size, an inverse modelling framework was developed in which the roles of input and output were reversed relative to the forward models. Particle size served as the sole predictor, and FRR, PLGA concentration, and channel width were each treated as separate regression targets. Four algorithms were selected for inverse prediction: linear regression, second-degree polynomial regression, gradient boosting regression (100 estimators), and support vector regression with a radial basis function kernel (*C* = 100, *γ* = 0.1). Each algorithm was independently trained for each of the three output variables, yielding twelve inverse models in total, each evaluated by *R*^2^ on a held-out 20% test set.

For inference, a user-specified target size was passed to each trained model to generate continuous predictions of the three process parameters. Because PLGA concentration and channel width are constrained to discrete experimental levels in practice, continuous predictions were post processed by rounding to the nearest permitted value: PLGA concentration was mapped to 2.5, 5.0, or 7.5 mg ml^−1^ and channel width to 100, 200, or 300 μm, while FRR predictions were retained as continuous values. This inverse prediction workflow allows candidate process conditions to be identified for a desired NP size without exhaustive experimental screening.

#### 2.5.5 Model evaluation metrics

Model performance was evaluated using three standard regression metrics: coefficient of determination *R*^2^, root mean squared error (RMSE), and mean absolute error (MAE).

The coefficient of determination was calculated as:

1 *R*^2^ = 1 − [∑(*y*_*i*_ − *ŷ*_*i*_)^2^/∑(*y*_*i*_ − *ȳ*)^2^].

where (*y*_*i*_) is the observed value, (*ŷ*_*i*_) is the predicted value, and (*ȳ*) is the mean observed value. Higher *R*^2^ values indicate better explanatory performance.2
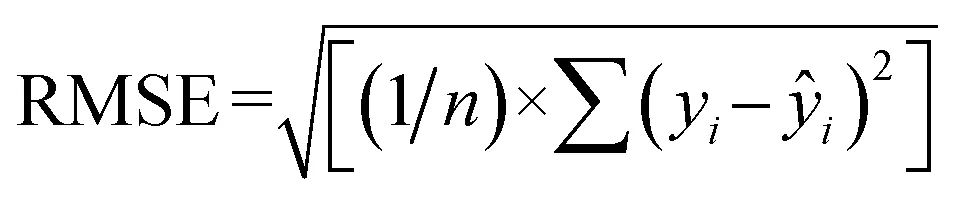

3 MAE = (1/*n*) × ∑|*y*_*i*_ − *ŷ*_*i*_|

## 3. Results and discussion

### 3.1 Effect of microchannel width on mixing dynamics and PLGA nanoparticle size

PLGA NPs were synthesized using microfluidic chips with channel widths of 100, 200, and 300 μm to investigate the influence of microchannel geometry on particle size. In all experiments, the organic phase consisted of 5 mg mL^−1^ PLGA dissolved in acetonitrile, while the aqueous phase comprised a 0.25% (w/v) PVA solution. The FRR, defined as the ratio of the organic phase flow rate (*Q*_c_) to the total aqueous phase flow rate (*Q*_s_), was maintained at 0.1 (*Q*_c_: 10 μL min^−1^/*Q*_s_: 100 μL min^−1^). The organic phase was introduced through the central inlet at a flow rate of 10 μL min^−1^, whereas the aqueous phase was supplied through the other inlet at 100 μL min^−1^ in which the aqueous phase was subsequently split into two side streams (flow rates of 50 μL min^−1^). The measured NP sizes and corresponding PDI values are summarized in Table S1.

As shown in Fig. 2a, increasing the channel width from 100 μm to 300 μm resulted in a systematic increase in NP size from 104.5 ± 1.45 nm to 153.7 ± 1.57 nm, with an intermediate size of 137.5 ± 0.5 nm obtained at a channel width of 200 μm. The low standard deviations observed under all conditions demonstrate the excellent reproducibility of the microfluidic synthesis process. Furthermore, all formulations exhibited PDI values below 0.3 (Table S1), indicating narrow particle size distributions and good colloidal quality. DLS measurements repeated after one week and one month showed no significant changes in either particle size or PDI, confirming the colloidal stability of the synthesized nanoparticles and the absence of aggregation during storage.

**Fig. 2:**
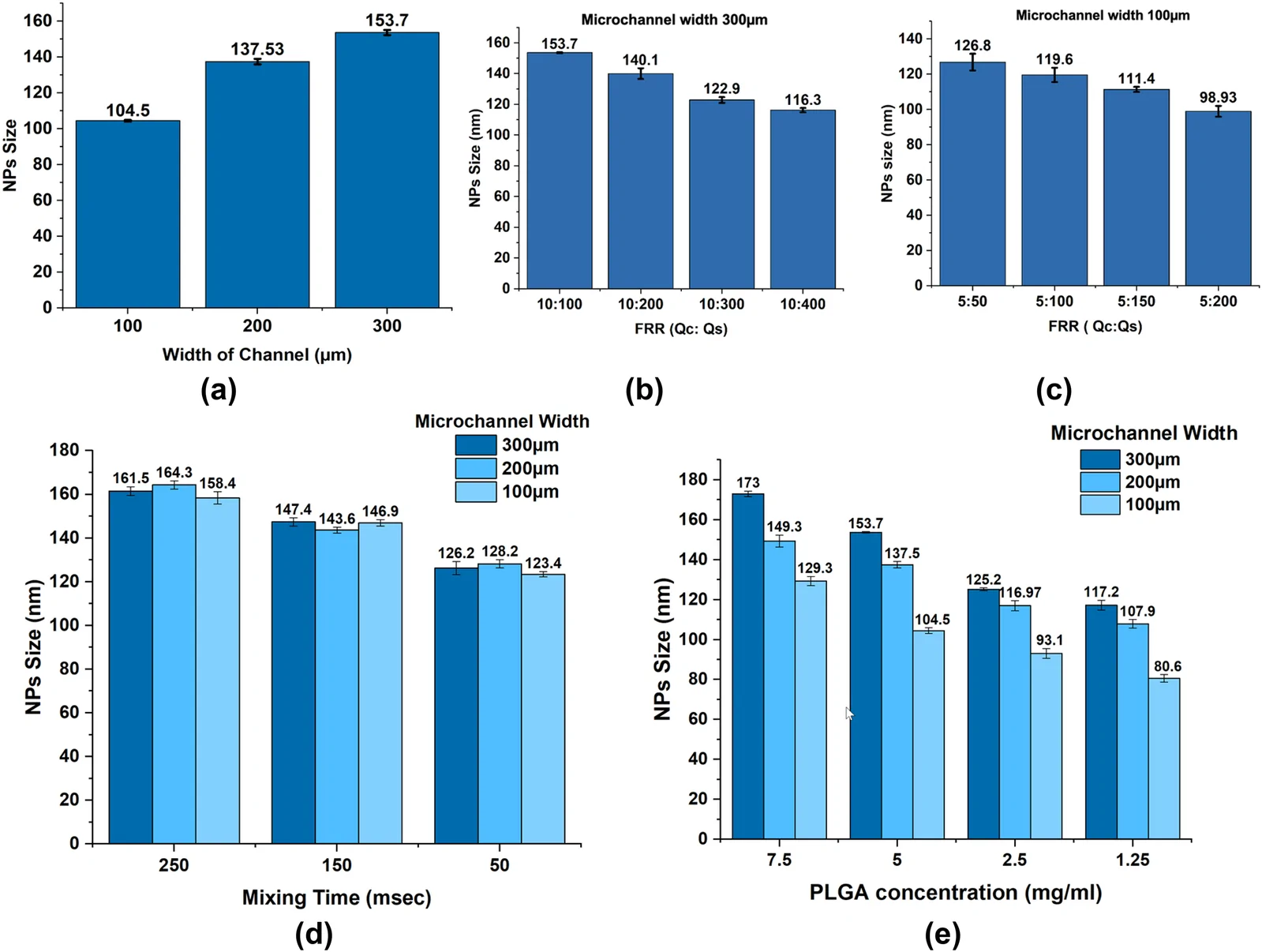
Influence of microfluidic operating conditions on PLGA NP size. (a) Effect of microchannel width on PLGA NP size. (b) Effect of FRR on PLGA NP size in a 300 μm microchannel. (c) Effect of FRR on PLGA NP size in a 100 μm microchannel. (d) Relationship between calculated mixing time and nanoparticle size for different microchannel widths. (e) The influence of polymer concentration on PLGA NPs at different microchannels widths (100, 200 and 300 μm).

The observed increase in NP size with channel width can be explained by changes in hydrodynamic focusing and mixing dynamics within the microfluidic device. In HFF systems, NP formation is governed by solvent-to-antisolvent mixing, which controls the rate of supersaturation development and the subsequent nucleation and growth of polymer aggregates. According to the model proposed by Karnik *et al.*,^30^ the characteristic mixing time can be estimated as:4
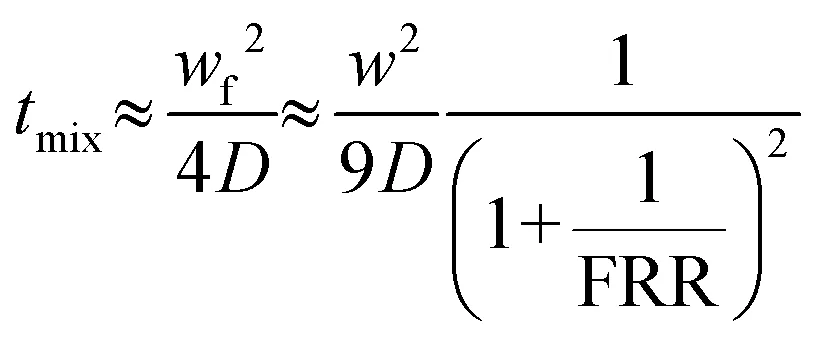
where *w*_f_ is the width of the focused stream, *w* is the microchannel width, *D* is the diffusivity of acetonitrile in the aqueous phase which is equal to 10^−9^ m^2^ s^−1^ and FRR is *Q*_c_/*Q*_s_ as defined above. This relationship indicates that the mixing time scales with the square of the channel width; therefore, increasing the channel width results in longer mixing times.

At a constant FRR, wider channels provide weaker hydrodynamic confinement of the organic stream, resulting in a broader focused stream and a longer diffusion path length for solvent exchange. Consequently, mixing occurs more slowly, leading to a slower rate of supersaturation development. Reduced supersaturation suppresses nucleation while allowing greater particle growth prior to stabilization, ultimately producing larger NPs. In contrast, narrower channels generate stronger hydrodynamic focusing, shorter diffusion distances, and faster solvent–antisolvent mixing. These conditions promote rapid supersaturation and nucleation while limiting particle growth, resulting in smaller NPs. Collectively, these results demonstrate that microchannel geometry can be used as an effective tool to regulate mixing behaviour and thereby control NP size during microfluidic synthesis.

### 3.2 Effect of flow rate ratio on mixing dynamics and PLGA nanoparticle size

The influence of FRR on NP formation during microfluidic nanoprecipitation has been extensively investigated in previous studies and is widely recognized as one of the most important parameters governing NP size.^9,31^ In the present work, these findings were verified using the microfluidic chip design employed throughout this study. PLGA NPs were synthesized using a 5 mg mL^−1^ PLGA solution in acetonitrile as the organic phase, which was introduced through the central inlet channel. The aqueous phase consisted of a 0.25% (w/v) PVA solution supplied through the second inlet, which subsequently bifurcated into two sheath streams upstream of the flow-focusing junction. To investigate whether the influence of FRR depended on channel geometry, the experiments were performed using microfluidic devices with channel widths of 100 and 300 μm. The measured PLGA NP sizes synthesized at different FRR and corresponding PDI values are summarized in Table S2.

As shown in Fig. 2b and c, decreasing the FRR resulted in a consistent decrease in NP size for both channel widths. For the 300 μm channel, the average NP size decreased from 153.7 ± 0.4 nm at FRR (*Q*_c_ : *Q*_s_) of 10 μL min^−1^ : 100 μL min^−1^ to 140.1 ± 3.5 nm, 122.9 ± 1.9 nm, and 116.3 ± 1.4 nm at FRRs of 10 μL min^−1^ : 200 μL min^−1^, 10 μL min^−1^ : 300 μL min^−1^, and 10 μL min^−1^ : 400 μL min^−1^, respectively. A similar trend was observed for the 100 μm channel, where particle size decreased from 126.8 ± 4.8 nm to 119.6 ± 4.1 nm, 111.4 ± 1.4 nm, and 98.9 ± 3.5 nm as the FRR values changed from 5 μL min^−1^ : 50 μL min^−1^ to 5 μL min^−1^ : 200 μL min^−1^.

These results also confirm that FRR is a critical parameter governing mixing dynamics and NP size, while also highlighting the important role of channel geometry in controlling PLGA nanoprecipitation within microfluidic flow-focusing systems.

### 3.3 Mixing time role in PLGA NP formation

To gain a deeper understanding of PLGA NP formation within the microfluidic channels and to determine whether mixing time is the primary parameter governing particle size, the mixing times associated with different channel widths and FRRs were calculated using eqn (4). According to this model, both FRR and microchannel width directly influence the characteristic mixing time and, consequently, the rate of solvent-to-antisolvent exchange during nanoprecipitation.

To further test the hypothesis that FRR and channel width influence NP formation primarily through their effect on mixing time, synthesis conditions were selected to achieve equivalent mixing times using different combinations of channel width and FRR. Mixing times of approximately 50, 150, and 250 ms were achieved under these conditions as mentioned in Table S3. As shown in Fig. 2, the mixing time model described in eqn (4) accurately captures the relationship between mixing time and NP size only when the influence of TFR is excluded. However, TFR itself can affect NP formation under otherwise identical process conditions. For example, NPs synthesized in a 100 μm microchannel at equivalent FRRs (*Q*_c_ : *Q*_s_ = 10 μL min^−1^ : 100 μL min^−1^ and 5 μL min^−1^ : 50 μL min^−1^) exhibited smaller particle size at higher TFR. The effect of TFR on mixing dynamics has been previously investigated by Saeed *et al.*^32^ Therefore, to decouple the impact of TFR on mixing dynamics, TFR was maintained constant at 110 μL min^−1^ to ensure comparable synthesis conditions across all mixing time comparison experiments as well as for the DoE and machine learning datasets discussed in subsequent sections.

The resulting NP sizes are shown in Fig. 2d. Remarkably, NPs synthesized under conditions yielding the same calculated mixing time exhibited very similar particle sizes. 123.4 ± 1.2, 128.2 ± 1.9, and 126.2 ± 3 nm were obtained for the 100, 200 and 300 μm microchannels when operated at FRRs of 0.27, 0.12, and 0.08, respectively. As calculated by eqn (4), these combinations of microchannel width and FRR correspond to the same mixing time. Similarly, at 150 ms, particle sizes of 146.9 ± 1.5, 143.6 ± 1.3 and 147.4 ± 1.9 nm were observed, while at 250 ms the corresponding sizes were 158.4 ± 2.8, 164.3 ± 1.8, and 161.5 ± 1.9 nm.

Quantitatively, the maximum deviation in mean particle size between different channel width and FRR combinations was 4.8 nm at 50 ms, 3.8 nm at 150 ms, and 5.9 nm at 250 ms, corresponding to relative differences of 3.8%, 2.6%, and 3.7%, respectively. The relative standard deviation (RSD) among these equivalent mixing time conditions remained ≤1.9% in all cases. These deviations were comparable to the standard deviation observed between replicate measurements under individual synthesis conditions (1.2–3.0 nm), indicating that the residual variation among different channel width and FRR combinations was within the range of experimental variability rather than arising from a systematic effect of channel width or FRR.

The close agreement in particle size across different microchannel widths and FRRs that produce the same mixing time demonstrates that mixing time is the primary parameter governing NP formation, whereas microchannel width and FRR influence particle size predominantly through their effect on the mixing process. The complete set of microchannel width–FRR combinations yielding identical mixing times, together with the corresponding NP size and PDI values, is provided in Table S3.

Furthermore, a clear positive correlation between mixing time and NP size can be observed in Fig. 2d. Increasing the mixing time from approximately 50 to 250 ms resulted in a progressive increase in PLGA NP diameter from approximately 125 nm to over 160 nm. These findings provide strong experimental evidence that mixing time serves as a unifying parameter linking microfluidic operating conditions to nanoparticle formation. Consequently, both FRR and channel width influence PLGA NP size primarily through their effect on mixing time, highlighting the importance of controlling microscale transport phenomena during hydrodynamic flow focusing nanoprecipitation.

### 3.4 Effect of PLGA concentration on nanoparticle size

The influence of polymer concentration on PLGA NP formation has also been widely reported in the literature and is recognized as an important factor affecting NP size.^9,33,34^ To investigate this effect within the present microfluidic setup, PLGA NPs were synthesized using PLGA concentrations of 1.25, 2.5, 5.0, and 7.5 mg mL^−1^. The flow conditions were maintained constant at an organic-to-aqueous flow rate ratio (*Q*_c_ : *Q*_s_) of 10 : 100, while experiments were performed using microchannels with widths of 100, 200, and 300 μm. NP characterization results are presented in Table S4.

As shown in Fig. 2e, particle size increased with increasing PLGA concentration across all channel widths investigated. This trend indicates a strong correlation between polymer concentration and the resulting NP size irrespective of the microchannel geometry. Although channel width influenced the absolute NP size, the effect of PLGA concentration remained consistent throughout all experimental conditions.

The increase in NP size with polymer concentration can be explained by the nanoprecipitation mechanism. During solvent-to-antisolvent mixing, higher PLGA concentrations result in a greater amount of polymer being available within the supersaturated region. Consequently, polymer rich nuclei formed during nucleation encounter a higher local polymer concentration, which promotes particle growth through polymer deposition and aggregation before stabilization. In addition, increasing the polymer concentration increases the probability of intermolecular interactions and collision events between polymer-rich domains, facilitating the formation of larger NPs.

From a kinetic perspective, higher PLGA concentrations lead to more rapid attainment of the critical supersaturation required for phase separation and reduce the characteristic aggregation time.^35^ As a result, particle growth becomes increasingly dominant relative to nucleation, yielding larger NPs. Conversely, at lower polymer concentrations, fewer polymer molecules are available for growth, resulting in smaller NPs with narrower size distributions. However, the effect of PLGA concentration should not be considered solely in terms of polymer availability. Increasing PLGA concentration may also modify the rheological properties of the organic phase, particularly its viscosity, which can affect solvent-to-antisolvent exchange and consequently the effective mixing time within the microchannel.^36^ Therefore, at the highest PLGA concentration investigated (7.5 mg mL^−1^), the actual mixing dynamics may deviate from those predicted by the simplified theoretical model in eqn (4) which assumes constant viscosity and concentration-independent transport properties. An increase in viscosity at high concentration leads to an increase in NP size.

The consistent trend observed across all channel widths suggests that polymer concentration acts independently of the geometric effects associated with channel width and mixing time. While channel geometry primarily influences NP formation through its effect on mixing dynamics, PLGA concentration directly determines the amount of polymer available for nucleation and growth and simultaneously affects the rheological properties of the system. Together, these results demonstrate that PLGA concentration is a key process parameter for controlling NP size during microfluidic nanoprecipitation and must be carefully optimized alongside FRR and channel geometry.

Future theoretical studies should incorporate organic phase concentration-dependent viscosity and diffusion coefficients, potentially coupled with CFD simulations, to more accurately predict mixing time and solvent displacement. This approach could improve the theoretical prediction of NP formation and size.

### 3.5 Design of experiments analysis and model development

A three-factor, three level Box–Behnken design (BBD) was employed to investigate the combined effects of three independent variables: flow rate ratio (FRR, range: 0.05–0.15), PLGA concentration (range: 2.5–7.5 mg mL^−1^), and microchannel width (range: 100–300 μm) on the size of PLGA NPs synthesized using the HFF microfluidic chips. A total of 17 experimental runs were performed, and the resulting NP sizes ranged from 93.4 to 171.9 nm (Table S5), confirming that these process variables strongly influenced NP formation.

Prior to model fitting, the fit summary (Table S6), lack of fit test (Table S7), and sequential model sum of squares analysis (Table S8) were evaluated to identify the most appropriate response surface model. The linear and 2FI models exhibited significant lack of fit (*p* = 0.0067 and *p* = 0.0075, respectively; Table S7), indicating that neither adequately captured the curvature in the response. The quadratic model significantly improved the fit (*F* = 22.43, *p* = 0.0006; Table S8), exhibited a non-significant lack of fit (*F* = 2.12, *p* = 0.2399; Table S7), and yielded the highest adjusted *R*^2^ (0.9952) and predicted *R*^2^ (0.9783) among the candidate models (Table S6). On this basis, the quadratic model was selected as the most appropriate representation of the experimental data and was subsequently used for ANOVA and NP size prediction.

The ANOVA results for the selected quadratic model are presented in Table S9. The model was highly significant (*F* = 373.40, *p* < 0.0001), confirming the suitability of the selected model for describing NP size within the investigated parameter space. Examination of the ANOVA results revealed that all three main factors significantly affected particle size (*p* < 0.0001). Microchannel width exhibited the largest *F*-value (1268.51), followed by PLGA concentration (1204.84) and FRR (781.29), indicating that channel geometry had the largest influence on NP size within the investigated parameter range. A similar trend was observed in the model sum of squares contributions, where microchannel width, PLGA concentration, and FRR contributed approximately 37.7%, 35.8%, and 23.3% of the model sum of squares, respectively, together accounting for nearly 97% of the variability captured by the model. These findings are consistent with the mechanistic analysis presented earlier, which demonstrated that channel width, PLGA concentration, and FRR directly influence solvent-to-antisolvent mixing and particle growth kinetics, thereby governing PLGA NP formation.

Among the interaction terms presented in Table S9, the interaction between PLGA concentration and microchannel width (BC) exhibited the highest statistical significance, with *F* = 30.02 and *p* = 0.0009. The FRR × microchannel width interaction (AC) showed a weaker but still significant effect (*F* = 6.94, *p* = 0.0337), whereas the FRR × PLGA concentration interaction (AB) was not statistically significant (*F* = 1.66, *p* = 0.2380). These results indicate that the effect of PLGA concentration on NP size is strongly dependent on microchannel width, while the interaction between FRR and PLGA concentration is negligible within the investigated range. This observation is consistent with the mixing time analysis described above, where channel width strongly influences solvent exchange dynamics and supersaturation development, thereby affecting the relative contribution of nucleation and particle growth. Consequently, PLGA concentration becomes more influential under slower mixing conditions associated with wider channels. The absence of a significant AB interaction further suggests that FRR and PLGA concentration primarily influence NP formation through distinct mechanisms. FRR primarily governs mixing dynamics and PLGA concentration affects polymer availability during NP formation.

The quadratic terms further revealed the presence of nonlinear behaviour. The quadratic effects of PLGA concentration (*B*^2^, *F* = 15.45, *p* = 0.0057) and microchannel width (*C*^2^, *F* = 44.90, *p* = 0.0003) were significant, indicating curvature in the response surface and suggesting that the influence of these factors on NP size was not strictly linear. Conversely, the quadratic effect of FRR (*A*^2^, *F* = 1.97, *p* = 0.2034) was not significant, indicating a largely linear response over the selected experimental range.

The statistical quality of the model was further confirmed by the fit statistics (Table S10). The model exhibited an excellent coefficient of determination (*R*^2^ = 0.9979), indicating that 99.79% of the variability in NP size was explained by the model. The adjusted *R*^2^ (0.9952) and predicted *R*^2^ (0.9783) were in close agreement, supporting strong predictive capability and minimal overfitting. The low coefficient of variation (C.V. = 1.17%) reflected high experimental precision, and the adequate precision value of 65.41 far exceeded the recommended threshold of 4, confirming an excellent signal to noise ratio and the suitability of the model for navigating the design space. The predictive capability of the model was further validated by the actual *versus* predicted plot (Fig. 3a), in which the experimental NP sizes closely followed the parity line with no apparent systematic deviations. The close agreement between measured and predicted values further confirmed the adequacy of the quadratic model for describing and predicting NP size within the investigated parameter space.

**Fig. 3:**
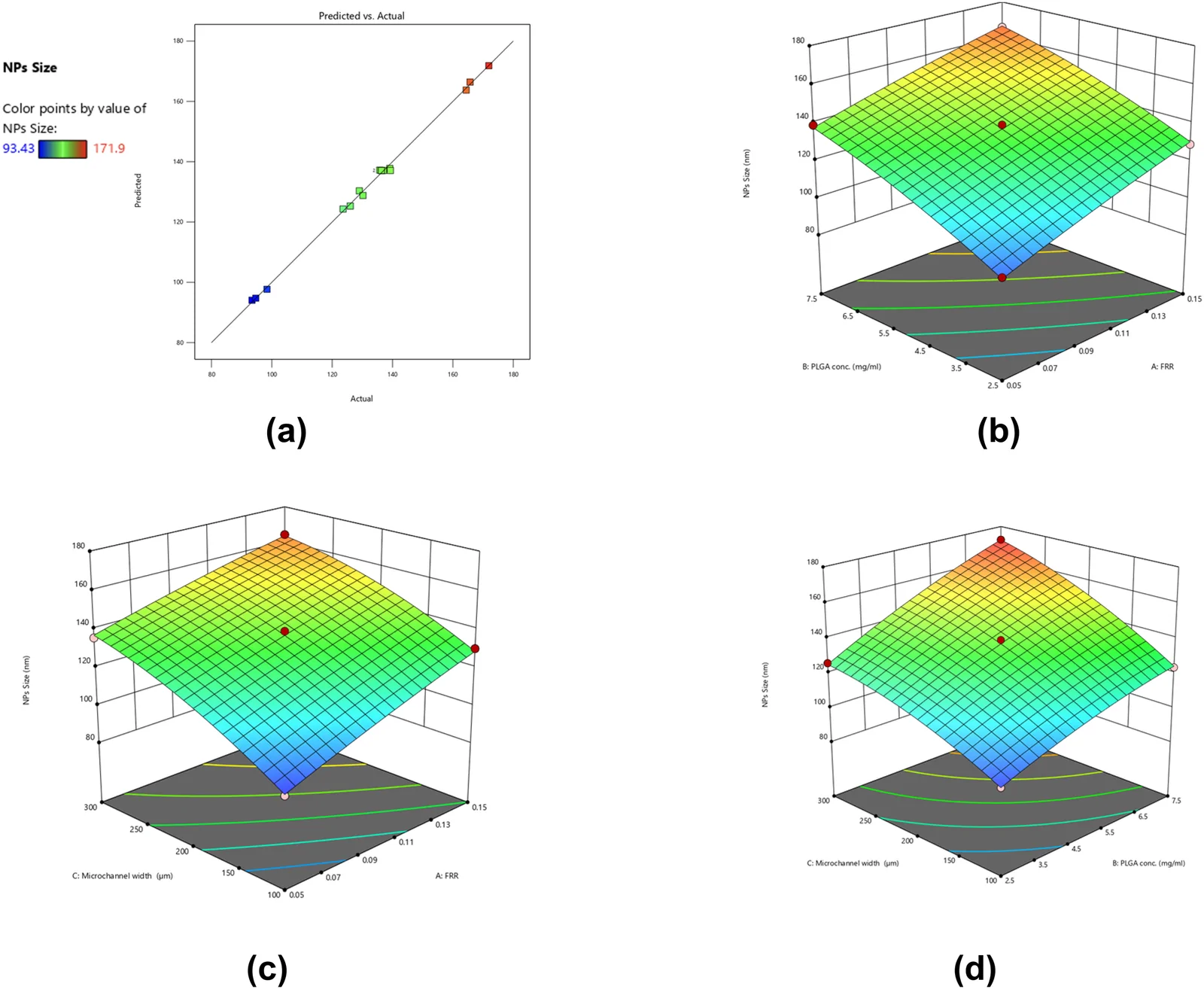
Statistical validation and response surface analysis of the Box–Behnken model for PLGA NP size prediction. (a) Predicted *versus* experimental NPs sizes obtained from the quadratic response surface model. (b) Three-dimensional response surface illustrating the combined effects of FRR and PLGA concentration on NP size at a fixed microchannel width. (c) Response surface showing the combined effects of FRR and microchannel width on NP size at a fixed PLGA concentration. (d) Response surface depicting the combined effects of PLGA concentration and microchannel width on NP size at a fixed FRR.

The final quadratic model in terms of coded factors was:

5 NP size = 137.08 + 15.32*A* + 19.03*B* + 19.52*C* − 1.00*AB* − 2.04*AC* + 4.25*BC* − 1.06*A*^2^ − 2.97*B*^2^ − 5.06*C*^2^

where *A*, *B*, and *C* represent FRR, PLGA concentration, and microchannel width, respectively. The positive coefficients of the linear terms indicate that increasing FRR, PLGA concentration, or microchannel width resulted in larger NPs within the investigated parameter space.

The response surface plots shown in Fig. 3b–d provide additional insight into the interactions between process variables. The FRR and PLGA concentration surface shown in Fig. 3b shows that NP size increased progressively with increasing PLGA concentration and FRR, with the highest particle sizes obtained at simultaneously high polymer concentrations and high FRR values. Similarly, the FRR and microchannel width surface (Fig. 3c) demonstrates a strong increase in NP size with increasing channel width, confirming the dominant role of microchannel geometry in controlling particle formation. Larger NPs were obtained when both polymer concentration and channel width were increased simultaneously (Fig. 3d), indicating a strong interaction between polymer availability and mixing dynamics.

The DoE analysis (Tables S6–S10) identified microchannel width as the dominant factor governing NP size, followed closely by PLGA concentration and FRR, while also revealing significant nonlinear effects and a strong interaction between polymer concentration and channel geometry. The excellent agreement between experimental observations, mechanistic mixing time analysis, and statistical modelling highlights the robustness of the developed predictive framework for PLGA NP synthesis.

### 3.6 Machine learning-based predictive model

#### 3.6.1 Comparative performance of machine learning models

The machine learning models were developed using the experimental dataset summarized in Table S11, which comprises PLGA NP synthesis conditions spanning three FRR values (0.05, 0.10, and 0.15) at constant TFR of 110 μL min^−1^, three PLGA concentrations (2.5, 5.0, and 7.5 mg mL^−1^), and three microchannel widths (100, 200, and 300 μm). The predictive performance of the evaluated machine learning models is summarized in Table S12 and Fig. 4a–c. As is evident from Table S12, gradient boosting, random forest, and polynomial (degree 2) are three top performance algorithms. Gradient boosting achieved the highest predictive accuracy, yielding the highest test (*R*^2^ = 0.9900) and the lowest prediction errors (RMSE = 2.65 nm and MAE = 2.13 nm). Random forest exhibited comparable performance (*R*^2^ = 0.9853, RMSE = 3.22 nm, MAE = 2.55 nm), followed closely by polynomial regression (degree 2) (*R*^2^ = 0.9754, RMSE = 4.17 nm, and MAE = 3.41 nm), whereas linear, ridge, and lasso regression produced comparable performance (*R*^2^ = 0.9688–0.9700).

The superior performance of gradient boosting and random forest indicates that NP size depends on nonlinear relationships and interactions among FRR, PLGA concentration, and microchannel width that are not fully captured by linear models. Overall, model performance followed the order: gradient boosting > random forest > polynomial regression > SVR > linear regression ≈ lasso regression ≈ ridge regression > decision tree.

#### 3.6.2 Model generalization and prediction accuracy

The train–test comparison of *R*^2^, RMSE, and MAE (Fig. 4a–c) demonstrated excellent generalization for gradient boosting, random forest, and SVR. Gradient boosting showed minimal performance degradation from training to testing (train/test) (*R*^2^: 0.9953/0.9900; RMSE: 1.79/2.65 nm; MAE: 1.51/2.13 nm), indicating negligible overfitting. Random forest (train/test) (*R*^2^: 0.9953/0.9853) and polynomial regression (degree 2) (0.9841/0.9754) exhibited similarly small increases in RMSE and MAE, confirming their robustness on unseen data.

**Fig. 4:**
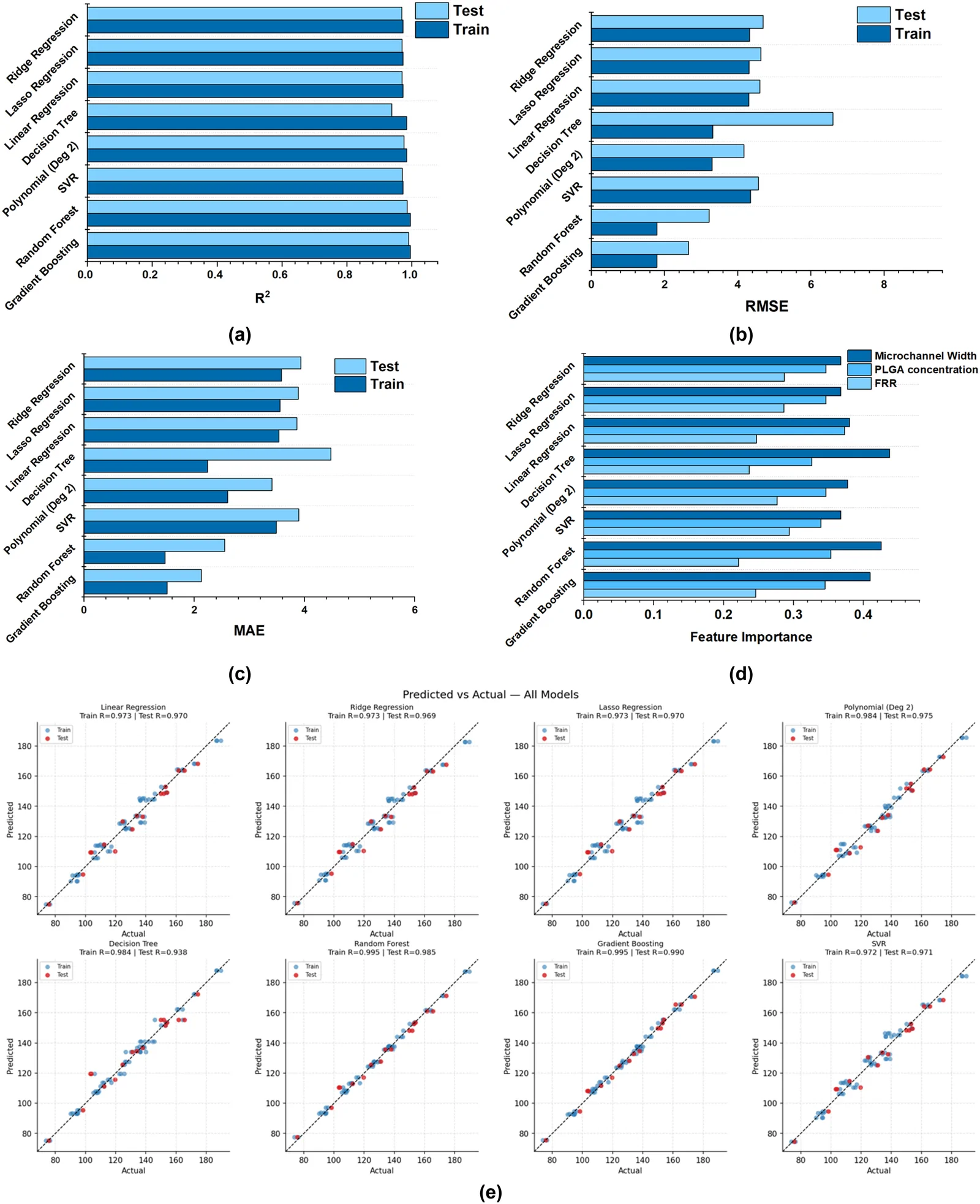
Comparative evaluation of machine learning models for predicting PLGA NP size. (a) Comparison of the *R*^2^ values. (b) Comparison of the corresponding RMSE values. (c) Comparison of MAE values for the training and test datasets. (d) Relative feature importance of the three process variables. (e) Parity plots comparing the experimentally measured and model-predicted NP sizes for the training and test datasets.

In contrast, the decision tree model exhibited the weakest generalization, with the largest decrease in *R*^2^ from 0.9839 to 0.9384 and the highest test RMSE (6.60 nm) and MAE (4.48 nm). This behaviour is characteristic of single decision trees, which are high variance learners and are more susceptible to overfitting, particularly for relatively small datasets (81 observations from 27 experimental conditions). Ensemble learning effectively mitigated this limitation, with random forest reducing variance through bootstrap aggregation and gradient boosting, further improving accuracy by sequentially correcting residual prediction errors.

The parity plots (Fig. 4e) further support these findings. For all models, the predicted NP sizes closely followed the 1 : 1 parity line, indicating good agreement with the experimental values. Gradient boosting exhibited the tightest clustering around the parity line for both training and test datasets, followed by random forest. Polynomial and linear regression models showed greater deviations, particularly at intermediate NP sizes, whereas the decision tree displayed the largest scatter, consistent with its lower predictive accuracy. Collectively, the parity plots corroborate the quantitative performance metrics and confirm that gradient boosting provided the most accurate and consistent predictions across the investigated size range.

The superior performance of gradient boosting can be attributed to its sequential ensemble learning strategy, in which successive decision trees iteratively minimize the residual errors of preceding models. Unlike random forest, which grows trees independently and aggregates their predictions to reduce variance, gradient boosting constructs trees sequentially, with each learner trained to minimize the residual errors of the preceding ensemble. This iterative error-correction strategy enables progressive refinement of complex nonlinear relationships among FRR, microchannel width, and PLGA concentration. In contrast, the second-order polynomial regression model is restricted to a predefined quadratic functional form and pairwise interaction terms, whereas gradient boosting adaptively captures nonlinear interactions without requiring their explicit specification. This enables the algorithm to effectively capture the nonlinear relationships and interactions among FRR, PLGA concentration, and microchannel width identified by the mechanistic and DoE analyses while maintaining excellent generalization for the relatively small experimental dataset.

#### 3.6.3 Cross validation (CV) performance

To further evaluate model robustness, 5-fold cross-validation was performed. The cross-validation metrics are summarized in Table S14 and compared in Fig. S1. Gradient boosting achieved the best overall performance, with the highest mean CV *R*^2^ of 0.9841 ± 0.0100 and the lowest CV RMSE (2.89 ± 0.40 nm) and CV MAE (2.42 ± 0.36 nm). Random forest ranked second (CV *R*^2^ = 0.9790 ± 0.0060), followed by polynomial regression (CV *R*^2^ = 0.9686 ± 0.0090). Linear, ridge, lasso, and SVR models exhibited comparable performance (CV *R*^2^ = 0.9599–0.9623), whereas decision tree showed the lowest predictive accuracy (CV *R*^2^ = 0.9511 ± 0.0335) and the highest prediction errors, indicating reduced robustness.

The mean cross-validation *R*^2^ of 0.9841 with a standard deviation of 0.0100 demonstrates that the gradient boosting model maintained consistently high predictive accuracy across the five validation folds. Compared with the single train–test split evaluation, the cross-validation performance remained similarly high, with only a slight reduction in *R*^2^, indicating that the model performance was not dependent on a particular data partition. The low standard deviation further confirms the stability and robustness of the model, suggesting minimal sensitivity to variations in the training and validation datasets and a low risk of overfitting.

The relatively small standard deviations observed for gradient boosting, random forest, and polynomial regression demonstrate stable predictive performance across the validation folds, whereas the larger variability of the decision tree reflects its higher sensitivity to the training data. These findings are consistent with the train–test evaluation and further confirm the superior robustness of the ensemble learning models.

The out-of-fold parity plots (Fig. S2) further validate these results. Gradient boosting exhibited the closest agreement between predicted and experimental NP sizes, followed by random forest, with both models showing data points tightly clustered around the 1 : 1 parity line. Polynomial regression also demonstrated good predictive capability, whereas the linear models, SVR, and particularly the decision tree exhibited slightly noticeable deviations from the parity line. Overall, the cross-validation analysis confirms that gradient boosting is the most robust and reliable model for NP size prediction within the range of parameters investigated in this study.

#### 3.6.4 Feature importance analysis

The relative importance of FRR, PLGA concentration, and microchannel width is presented in Fig. 4d and Table S13. Despite differences in the underlying algorithms, all models consistently identified the same ranking of predictor importance, which is microchannel width > PLGA concentration > FRR, and it is consistent with the *F* values obtained from DoE analysis based on ANOVA *F*-values and percentage contributions. It should be noted, however, that the ANOVA percentage contributions and ML feature importance values quantify different statistical concepts and therefore are not directly comparable numerically. The DoE percentage contributions represent the proportion of response variance explained by each factor within the experimental design, whereas the ML feature importance values reflect the relative contribution of each variable to the predictive performance of a specific learning algorithm.

Microchannel width was the dominant predictor across all models, with normalized importance values ranging from 0.367 to 0.437, followed by PLGA concentration (0.326–0.353) and FRR (0.221–0.294). The observed variation in feature importance among the ML models reflects differences in their underlying learning and feature attribution mechanisms rather than differences in the physical influence of the process variables. Tree-based models, particularly decision tree (0.437), random forest (0.425), and gradient boosting (0.409), assigned the highest importance to microchannel width because their importance scores are derived from cumulative reductions in node impurity. In contrast, the importance values for linear, polynomial, and kernel-based models were obtained using model-specific or model-agnostic feature attribution methods, which evaluate predictor influence differently. Therefore, variations in the absolute importance values across models are expected and should not be interpreted as contradictory results.

The consistent feature ranking across linear, polynomial, kernel-based, and ensemble learning algorithms demonstrates that the relative influence of the process variables is robust and independent of the modelling approach. The predominance of microchannel width reflects its direct influence on microfluidic mixing and solvent diffusion during NP formation, while PLGA concentration contributes through its effects on polymer availability and solution viscosity. In contrast, FRR exhibited relatively low importance within the investigated design space, indicating a comparatively smaller influence on NP size as compared to channel width and PLGA concentration. Therefore, although the DoE and ML analyses quantify variable importance using different mathematical frameworks, both consistently identify the same hierarchy of process parameters and support the same physical interpretation. The DoE analysis explains the contribution of each factor to the experimental response variance, whereas the ML analysis confirms that the same variables dominate predictive performance across multiple independent learning algorithms, providing complementary evidence for the robustness of the identified process response relationships.

### 3.7 Experimental validation of DoE and machine learning models by inverse design

To further validate the predictive capability of the developed DoE and ML models, inverse design experiments were performed to identify the process conditions required to achieve predefined NP sizes. Target NP sizes of 80, 120, and 160 nm were specified, and the corresponding process parameters, FRR, PLGA concentration, and microchannel width (*w*), were determined using the quadratic DoE model (eqn (6)) and the developed ML models. Importantly, the inverse design predictions were constrained to experimentally feasible process conditions. FRR was retained as a continuous variable because it can be precisely adjusted by tuning the organic and aqueous flow rates using syringe pumps. In contrast, microchannel width was constrained to the fabricated device dimensions of 100, 200, and 300 μm, as intermediate channel widths such as 237 μm could not be physically tested without fabricating a new device. PLGA concentration was also restricted to the experimentally investigated levels of 2.5, 5.0, and 7.5 mg mL^−1^. Therefore, any continuous ML predicted values for PLGA concentration and microchannel width were mapped to the nearest experimentally available level before synthesis. The predicted process conditions were subsequently implemented experimentally to synthesize PLGA NPs.

6 NP size = −2.21 + 512.925 × FRR + 9.7645 × PLGA conc. + 0.353625 × *w* − 8.00 × FRR × PLGA conc. − 0.4085 × FRR × *w* + 0.01699 × PLGA conc. × *w* − 424 × FRR^2^ − 0.4752 × PLGA conc.^2^ − 0.000506 × *w*^2^

For ML-based inverse design, all eight machine learning models were initially used to predict the process parameters required to achieve the target NP sizes, and the complete sets of predicted conditions for DoE and ML are provided in Tables S15–S18. For experimental validation, four representative models (gradient boosting, support vector regression (SVR), polynomial regression, and linear regression) were selected based on their predictive performance, algorithmic diversity, and the close agreement of the predicted process conditions among models within the same algorithmic class. For example, tree based models (gradient boosting, random forest, and decision tree) predicted very similar process parameters for a given target NP size, making experimental evaluation of all three models unnecessary. The predictive capability of the selected models was subsequently assessed by experimentally synthesizing PLGA NPs under the predicted FRR, PLGA concentration, and microchannel width and comparing the resulting NP sizes with the corresponding target values.

The experimentally obtained NP sizes are presented in Fig. 5. Overall, both the DoE and the ML models successfully identified process conditions capable of producing NPs close to the desired target sizes of 80, 120, and 160 nm, although accuracy varied among models. It should be noted that parameter discretization introduces minimal prediction deviation, as demonstrated by the close agreement between predicted and experimentally obtained particle sizes after rounding of formulation and device parameters. The deviation from the target particle size was relatively small, despite the limited number of discrete channel width and PLGA concentration values available. More refined inverse design could be achieved in future studies by fabricating chips with smaller increments in channel width or by employing microfabrication technologies that allow continuous control of channel geometry and concentration of organic phase, thereby reducing discretization-related deviations.

**Fig. 5:**
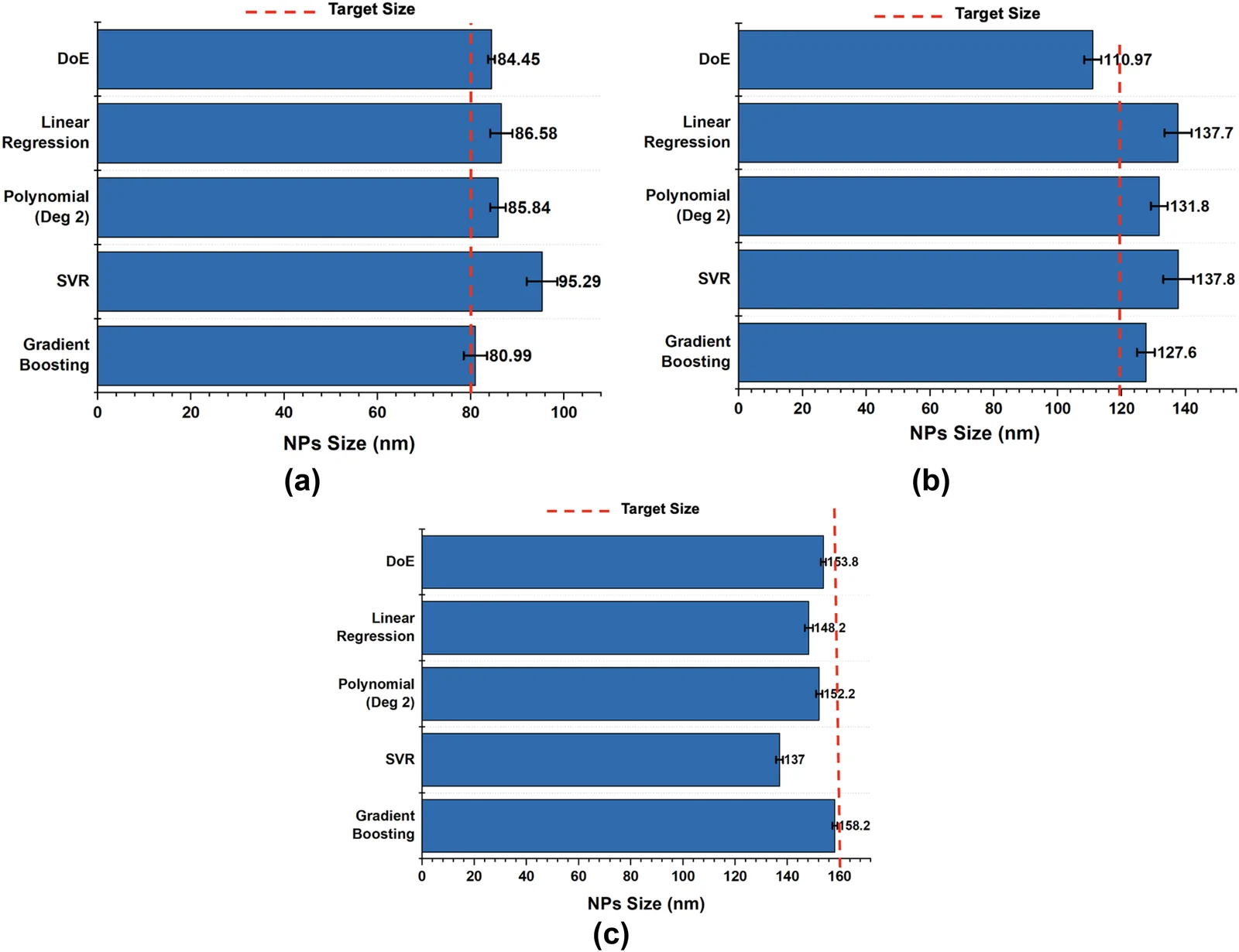
Experimental validation of the inverse-design capability of the DoE and selected ML models for synthesizing PLGA NPs with predefined target sizes: (a) 80 nm, (b) 120 nm, and (c) 160 nm.

The gradient boosting model consistently produced NP sizes in closest agreement with the target values across all three inverse-design experiments, demonstrating the highest accuracy overall. Other ML models and DoE showed more pronounced, random deviations from the target sizes, still reflecting their limited ability to capture the nonlinear relationships governing NP formation.

The superior performance of the gradient boosting model is consistent with its quantitative evaluation, which showed the highest test *R*^2^ and the lowest RMSE and MAE among all investigated models. The close agreement between predicted and experimentally obtained NP sizes confirms that the model effectively captured the nonlinear relationships among FRR, PLGA concentration, and microchannel width.

Although the inverse design experiments demonstrated the capability of the developed models to achieve predefined PLGA NP sizes, their application for the development of precisely controlled nanomedicines across the full clinically relevant size range (20–200 nm) requires further validation. The current models were trained and validated within a limited design space, with experimental verification performed only at three target sizes (80, 120, and 160 nm).

To further assess whether incorporating physical knowledge could improve the predictive performance, we evaluated two physics-informed neural network (PINN) variants using our current experimental dataset. The baseline model used FRR and channel width, while the physics-feature model additionally used the analytically calculated mixing time from eqn (4). The PINN-like model incorporated the same relation as a regularization constraint. As shown in Fig. S3 and S4, the physics-feature model generally provided more consistent improvements in held-out RMSE, particularly under extrapolation conditions, whereas the PINN-like approach did not consistently outperform the other models. These results indicate that the analytical mixing time relation provides a useful physics-based inductive bias. As discussed in detail in section 3.4, concentration plays an important role in mixing time and NP formation; therefore, future work should incorporate concentration explicitly into the governing equation to further improve model performance and generalization.

## 4. Conclusion

This work establishes a unified mechanistic, statistical, and machine learning framework for the prediction and inverse design of PLGA NP synthesis in HFF microfluidics. Systematic experiments demonstrated that microchannel width, FRR, and PLGA concentration collectively govern NP formation, with mixing time serving as the key mechanistic parameter linking microfluidic transport phenomena to NP size. NPs synthesized under different operating conditions but equivalent mixing times exhibited nearly identical sizes, confirming the dominant role of mixing dynamics during nanoprecipitation.

The quadratic DoE model accurately described the experimental data and identified microchannel width as the most influential process variable, followed by PLGA concentration and FRR. Complementary machine learning analysis further improved predictive performance, with ensemble learning models outperforming other regression models. Among the evaluated algorithms, gradient boosting consistently achieved the highest predictive accuracy, strongest generalization, and best cross-validation performance. Importantly, feature-importance analysis from all machine learning models closely agreed with the DoE findings, providing confidence in both the statistical and the physical interpretation of the results.

The predictive capability of the developed models was further demonstrated through inverse-design experiments, in which predefined NP sizes of 80, 120, and 160 nm were successfully translated into experimentally implementable synthesis conditions. The close agreement between experimentally obtained and target particle sizes confirms that machine learning, particularly gradient boosting, can reliably identify process parameters for producing NPs with user-defined characteristics due to its ability to accurately capture the nonlinear interactions among FRR, PLGA concentration, and microchannel width identified by the mechanistic and DoE analyses. This capability represents a significant advance beyond conventional trial-and-error optimization and establishes a practical route toward intelligent, data-driven microfluidic manufacturing. In inverse design, FRR, which can be easily adjusted at line, was treated as a continuous variable, while PLGA concentration and microchannel width, which are more difficult to change during operation, were treated as discrete variables. For reproducible mass manufacturing, custom fabricated chips with predefined channel dimensions could be used, while for small scale and tailored manufacturing, future model training could focus more on easily adjustable at-line variables such as FRR. The integration of upstream mixing could also allow PLGA concentration to be adjusted at-line, providing greater flexibility in future applications.

Overall, this study demonstrates that integrating mechanistic understanding with DoE and machine learning enables accurate prediction, optimization, and inverse design of PLGA NP synthesis within the investigated experimental domain. However, the current dataset is limited to the PLGA compositions, molecular characteristics, and operating conditions investigated in this study, and therefore the generalizability of the developed models to different PLGA molecular weights, end groups, other biodegradable polymers, and extreme NP size ranges remains to be established. Future studies should expand the experimental parameter space by incorporating PLGA with different molecular weights and end groups, other biodegradable polymers such as PLA and PCL, and broader FRR ranges. Such expanded datasets would provide a stronger basis for evaluating model transferability and developing more generalizable predictive and inverse-design frameworks for microfluidic NP synthesis.

## Author contributions

All authors have approved the final version of the manuscript.

## Conflicts of interest

There are no conflicts to declare.

## Supplementary Material

LC-026-D6LC00612D-s001

## Data Availability

The data supporting this article have been included as part of the supplementary information (SI). Supplementary information is available. See DOI: https://doi.org/10.1039/d6lc00612d. Code and dataset are publicly available at https://github.com/muhammadsaeed3-gif/data-driven-modeling-of-plga-nps.
