# Supplementary material for: Data-driven and mechanistic modelling of PLGA nanoparticle formation in hydrodynamic flow focusing microfluidics
Source: Lab Chip. 2026 Sep 21;26(21):5837–52. doi: 10.1039/d6lc00612d (PMC13644315; doi:10.1039/d6lc00612d)
Supplement: Supplementary file 1 [file LC-026-D6LC00612D-s001.pdf]

Supplementary Information (SI) for Lab on a Chip.
This journal is © The Royal Society of Chemistry 2026

Supporting Information

Data Driven and Mechanistic Modelling of PLGA Nanoparticle Formation in Hydrodynamic Flow-Focusing Microfluidics

Muhammad Mubashar Saeed[1-4 *], Muhammad Turab[5], Lasse H. E. Thamdrup[6], Anja Boisen[6], Eadaoin Carthy [2-4], Nicholas Dunne[2,3,6-11], David Kinahan [2-4,8]
Corresponding Author email: Muhammad.saeed3@mail.dcu.ie

1ML-Labs Centre for Research Training, Dublin City University, Dublin, Ireland
2School of Mechanical and Manufacturing Engineering, Dublin City University, Dublin, Ireland
3Biodesign Europe, Dublin City University, Dublin, Ireland
4RAPID Institute, Dublin City University, Dublin, Ireland
5School of Computing, Dublin City University, Dublin, Ireland
6 Drug delivery and Sensing (IDUN) section, Department of Health Technology, Technical University of Denmark, Ørsteds Plads 345C, 2800 Kongens Lyngby, Denmark
7Centre for Medical Engineering Research, Dublin City University, Dublin, Ireland
8Advanced Manufacturing Research Centre (I-Form), School of Mechanical and Manufacturing Engineering, Dublin City University, Dublin, Ireland
9School of Pharmacy, Queen's University Belfast, Belfast, UK
10Advanced Processing Technology Research Centre, Dublin City University, Dublin, Ireland
11Research Centre for Medical Devices (CÚRAM), Biomedical Sciences, University of Galway, Galway, Ireland

Table S1: Effect of microchannel width on the size and PDI of PLGA NPs

Microchannel width (µm)	PLGA NPs size (nm)	PDI
100	104.5 ± 1.45	0.16 ± 0.008
200	137.5 ± 0.5	0.05 ± 0.01
300	153.7 ± 1.57	0.07 0.03

Table S2: Effect of FRR on the size and PDI of PLGA NPs

Microchannel width (µm)	FRR ($Q_c : Q_s$)	PLGA NPs size (nm)	PDI
100	5:200	98.93±3.1	0.24±0.03
100	5:150	111.4±1.42	0.16±0.01
100	5:100	119.6±4.1	0.16±0.01
100	5:50	126.8±4.8	0.1±0.02
300	10:400	116.3±1.4	0.12±0.01
300	10:300	122.9±1.9	0.09±0.02
300	10:200	140.1±3.5	0.04±0.019
300	10:100	153.7±0.4	0.07±0.03

Table S3: PLGA NPs size and PDI) synthesized at different FRR and microchannel widths while maintaining identical mixing time

Mixing time	Microchannel width (µm)	FRR ($Q_c : Q_s$)	PLGA NPs size (nm)	PDI
50	100	23.33: 86.67	123.4±1.2	0.06±0.01
50	300	11.67: 98.33	128.2±1.9	0.07±0.01
50	300	7.78: 102.22	126.2±3	0.05±0.03
150	100	40.42: 69.58	146.9±1.5	0.04±0.005

150	200	20.21: 89.79	143.6±1.3	0.03±0.02
150	300	13.47: 96.53	147.4±1.9	0.04±0.03
250	100	52.28: 57.72	158.4±2.8	0.02±0.01
250	200	26.06:83.94	164.3±1.8	0.04±0.02
250	300	17.43:92.57	161.5±1.9	0.03±0.02

Table S4: Effect of PLGA concentration on the size and PDI of PLGA NPs

**Microchannel width (μm)**	**PLGA conc. (mg/ml)**	**PLGA NPs size (nm)**	**PDI**
100	1.25	80.6±1.9	0.18±0.01
100	2.5	93.05±2.4	0.13±0.02
100	5	104.5±1.4	0.16±0.01
100	7.5	129.3±2.3	0.1±0.03
200	1.25	107.9±2.2	0.18±0.02
200	2.5	116.7±2.5	0.12±0.01
200	5	137.5±1.6	0.05±0.01
200	7.5	149.3±3	0.06±0.01
300	1.25	117.2±2.5	0.26±0.01
300	2.5	125.2±0.7	0.1±0.01
300	5	153.7±0.4	0.07±0.03
300	7.5	173±1.3	0.06±0.01

Table S5: Box–Behnken experimental design matrix and corresponding PLGA NPs sizes obtained under different synthesis conditions

**std**	**Run**	**Factor 1 (FRR: A)**	**Factor 2 (PLGA concentration: B) mg/ml**	**Factor 3 (Microchannel width : C) μm**	**PLGA NPs Size (nm)**
12	1	0.1	7.5	300	171.9
17	2	0.1	5	200	136.3
7	3	0.05	5	300	135.8
14	4	0.1	5	200	136.5
4	5	0.15	7.5	200	165.7
1	6	0.05	2.5	200	98.4
13	7	0.1	5	200	136.3
8	8	0.15	5	300	164.4
3	9	0.05	7.5	200	139.1
6	10	0.15	5	100	130.2
5	11	0.05	5	100	93.43
10	12	0.1	7.5	100	123.6
2	13	0.15	2.5	200	129
9	14	0.1	2.5	100	94.69
16	15	0.1	5	200	137
11	16	0.1	2.5	300	126
15	17	0.1	5	200	139.3

Table S6: Fit Summary Response NPs size

**Source**	**Sequential p value**	**Lack of Fit p value**	**Adjusted R^2**	**Predicted R^2**	**Remark**
Linear	< 0.0001	0.0067	0.9587	0.9415	
2FI	0.2232	0.0075	0.9647	0.9352	
Quadratic	0.0006	0.2399	0.9952	0.9783	**Suggested**
Cubic	0.2399		0.9968		Aliased

Table S7: Lack of Fit tests

**Source**	**Sum of**	**df**	**Mean**	**F-value**	**p-value**	**Remark**

	Squares		Square			
Linear	264.94	9	29.44	18.15	0.0067	
2FI	172.08	6	28.68	17.68	0.0075	
Quadratic	10.34	3	3.45	2.12	0.2399	**Suggested**
Cubic	0.0000	0				Aliased
Pure Error	6.49	4	1.62			

Table S8: Sequential Model Sum of Squares

Source	Sum of Squares	df	Mean Square	F-value	p-value	Remark
**Mean vs Total**	2.998E+05	1	2.998E+05			
**Linear vs Mean**	7822.94	3	2607.65	124.89	< 0.0001	
**2FI vs Linear**	92.85	3	30.95	1.73	0.2232	
**Quadratic vs 2FI**	161.76	3	53.92	22.43	0.0006	**Suggested**
**Cubic vs Quadratic**	10.34	3	3.45	2.12	0.2399	Aliased
**Residual**	6.49	4	1.62			
**Total**	3.079E+05	17	18112.29			

Table S9: ANOVA for Quadratic model

Source	Sum of Squares	df	Mean Square	F-value	p-value	Remark
**Model**	8077.53	9	897.50	373.40	< 0.0001	**significant**
**A-FRR**	1877.93	1	1877.93	781.29	< 0.0001	
**B-PLGA conc.**	2895.99	1	2895.99	1204.84	< 0.0001	
**C-Microchannel width**	3049.02	1	3049.02	1268.51	< 0.0001	
**AB**	4.00	1	4.00	1.66	0.2380	
**AC**	16.69	1	16.69	6.94	0.0337	
**BC**	72.17	1	72.17	30.02	0.0009	
**A^2**	4.73	1	4.73	1.97	0.2034	
**B^2**	37.14	1	37.14	15.45	0.0057	
**C^2**	107.91	1	107.91	44.90	0.0003	
**Residual**	16.83	7	2.40			
**Lack of Fit**	10.34	3	3.45	2.12	0.2399	**not significant**
**Pure Error**	6.49	4	1.62			

Table S10: Fit statistics

**Std. Dev.**	1.55	**R^2**	0.9979
**Mean**	132.80	**Adjusted R^2**	0.9952
**C.V. %**	1.17	**Predicted R^2**	0.9783
		**Adeq Precision**	65.4060

Table S11: Data set for PLGA NPs synthesized at different FRR(0.05,0.1and 0.15), PLGA conc. (2.5, 5, and 7.5 mg/ml) and microchannel widths (100, 200, and 300 µm)

FRR	PLGA Conc. (mg/ml)	Microchannel width (µm)	Size (nm)	PDI
0.05	7.5	300	153.1	0.05523
0.05	7.5	300	150.2	0.04
0.05	7.5	300	152.9	0.05563
0.1	7.5	300	171.9	0.064
0.1	7.5	300	174.5	0.06017
0.1	7.5	300	172.6	0.0496
0.15	7.5	300	189.8	0.02
0.15	7.5	300	186.8	0.03273
0.15	7.5	300	187.2	0.09922
0.05	5	300	135.8	0.08421
0.05	5	300	134.1	0.05452
0.05	5	300	133.2	0.08591
0.1	5	300	154.1	0.09432
0.1	5	300	153.3	0.0356
0.1	5	300	153.7	0.09432
0.15	5	300	164.4	0.03761

0.15	5	300	160.7	0.03589
0.15	5	300	161.5	0.03291
0.05	2.5	300	112.4	0.24
0.05	2.5	300	110	0.242
0.05	2.5	300	112.3	0.241
0.1	2.5	300	126	0.108
0.1	2.5	300	124.6	0.09492
0.1	2.5	300	125.1	0.09928
0.15	2.5	300	137.9	0.05733
0.15	2.5	300	138.9	0.078
0.15	2.5	300	135.9	0.09898
0.05	7.5	200	139.1	0.075
0.05	7.5	200	137.9	0.02775
0.05	7.5	200	134.2	0.06
0.1	7.5	200	151.9	0.06381
0.1	7.5	200	150	0.05039
0.1	7.5	200	146	0.07
0.15	7.5	200	165.7	0.075
0.15	7.5	200	164.5	0.05
0.15	7.5	200	161.8	0.03859
0.05	5	200	108.9	0.1007
0.05	5	200	106.7	0.1283
0.05	5	200	108	0.1299
0.1	5	200	136.3	0.039
0.1	5	200	137	0.04057
0.1	5	200	139.3	0.0598
0.15	5	200	144.6	0.06442
0.15	5	200	145.7	0.09615
0.15	5	200	141.9	0.08774
0.05	2.5	200	98.4	0.2012
0.05	2.5	200	94.66	0.1969
0.05	2.5	200	96.07	0.1787
0.1	2.5	200	114.9	0.1095
0.1	2.5	200	119.7	0.1371
0.1	2.5	200	116.3	0.1237
0.15	2.5	200	129	0.1086
0.15	2.5	200	127	0.1314
0.15	2.5	200	126.2	0.073
0.05	7.5	100	111.3	0.1406
0.05	7.5	100	117.2	0.1162
0.05	7.5	100	112.3	0.1046
0.1	7.5	100	123.6	0.07622
0.1	7.5	100	125.8	0.07062
0.1	7.5	100	122.5	0.06781
0.15	7.5	100	140.1	0.087
0.15	7.5	100	136.3	0.07266
0.15	7.5	100	136.5	0.00599
0.05	5	100	93.43	0.1568
0.05	5	100	91.44	0.1616
0.05	5	100	94.93	0.12
0.1	5	100	106.1	0.1638
0.1	5	100	104	0.1718
0.1	5	100	103.3	0.1539
0.15	5	100	130.2	0.1087
0.15	5	100	131.1	0.1272
0.15	5	100	126.7	0.07194
0.05	2.5	100	73.84	0.2178
0.05	2.5	100	75.78	0.2081
0.05	2.5	100	76.52	0.2124
0.1	2.5	100	94.69	0.1161
0.1	2.5	100	94.2	0.1295
0.1	2.5	100	90.27	0.154
0.15	2.5	100	105.3	0.1396
0.15	2.5	100	108.2	0.11

0.15	2.5	100	107.1	0.1193

Table S12 Training and test performance metrics, including the coefficient of determination (R^2), root mean square error (RMSE), mean square error (MSE), and mean absolute error (MAE), for the evaluated machine learning models used to predict PLGA NPs size.

Model	Train R^2	Test R^2	Train RMSE	Test RMSE	Train MSE	Test MSE	Train MAE	Test MAE
**Gradient Boosting**	0.9953	0.9900	1.789	2.6538	3.2015	7.0429	1.5069	2.1292
**Random Forest**	0.9953	0.9853	1.7904	3.2191	3.2056	10.3628	1.4699	2.5519
**Polynomial (Deg 2)**	0.9841	0.9754	3.3011	4.1662	10.8974	17.3574	2.6040	3.4094
**SVR**	0.9724	0.9705	4.3463	4.5644	18.8902	20.8342	3.4899	3.8944
**Linear Regression**	0.9729	0.9699	4.3066	4.6033	18.5471	21.1902	3.5342	3.8619
**Lasso Regression**	0.9728	0.9694	4.3101	4.6349	18.5776	21.4824	3.5552	3.8869
**Ridge Regression**	0.9726	0.9688	4.3255	4.6919	18.7096	22.0141	3.5830	3.9334
**Decision Tree**	0.9839	0.9383	3.3154	6.5984	10.9919	43.5384	2.2419	4.4791

Table S13 Normalized feature importance of the input variables for PLGA NPs size prediction obtained from the evaluated machine learning models

Model	FRR	PLGA conc.	width
**Linear Regression**	0.2866	0.3463	0.3671
**Ridge Regression**	0.2866	0.3459	0.3674
**Lasso Regression**	0.2863	0.3462	0.3675
**Polynomial (Deg 2)**	0.2764	0.3462	0.37730
**Decision Tree**	0.2366	0.3259	0.4374
**Random Forest**	0.2214	0.3531	0.4254
**Gradient Boosting**	0.2458	0.3448	0.4093
**SVR**	0.2936	0.3390	0.3673

Table S14: Five-fold cross-validation (CV) performance metrics of the evaluated machine learning models for PLGA NPs size prediction

Model	CV R^2 (mean)	CV R^2 (std)	CV RMSE (mean)	CV RMSE (std)	CV MAE (mean)	CV MAE (std)
**Gradient Boosting**	0.9841	0.01	2.8908	0.4047	2.4178	0.3649
**Random Forest**	0.9790	0.006	3.5819	0.7247	2.9006	0.5348
**Polynomial (Deg 2)**	0.9686	0.009	4.3227	0.3825	3.3564	0.2425
**Ridge Regression**	0.9623	0.018	4.6168	0.3116	3.8577	0.1901
**Lasso Regression**	0.9622	0.019	4.5980	0.3193	3.8363	0.1542
**Linear Regression**	0.9620	0.0199	4.5862	0.3460	3.8268	0.1629
**SVR**	0.9599	0.022	4.6871	0.4619	3.9195	0.2719
**Decision Tree**	0.9511	0.0335	5.1089	1.4015	3.8960	1.0129

Table S15: Optimized DoE derived process parameters for the inverse design of PLGA nanoparticles with target sizes of 80, 120, and 160 nm

Target NP size	FRR	PLGA conc.	Microchannel width

		(mg/ml)	(µm)
**80 nm**	0.062	100	2.5
**120 nm**	0.122	100	5
**160 nm**	0.134	300	5

Table S16: Process parameters predicted by different machine learning models for the inverse design of PLGA nanoparticles with a target size of 80 nm

Model	FRR	PLGA conc. (mg/ml)	Microchannel width (µm)
**Gradient Boosting**	0.0529	2.5	100
**Random Forest**	0.0555	2.5	100
**Polynomial (Deg 2)**	0.0553	2.5	100
**Ridge Regression**	0.0644	2.5	100
**Lasso Regression**	0.1023	2.5	100
**Linear Regression**	0.0638	2.5	100
**SVR**	0.1	2.5	100
**Decision Tree**	0.05	2.5	100

Table S17: Process parameters predicted by different machine learning models for the inverse design of PLGA nanoparticles with a target size of 120 nm

Model	FRR	PLGA conc. (mg/ml)	Microchannel width (µm)
**Gradient Boosting**	0.1001	7.5	100
**Random Forest**	0.0900	7.5	100
**Polynomial (Deg 2)**	0.0978	5	200
**Ridge Regression**	0.0956	5	200
**Lasso Regression**	0.1023	5	200
**Linear Regression**	0.0955	5	200
**SVR**	0.1	5	200
**Decision Tree**	0.1250	5	100

Table S18: Process parameters predicted by different machine learning models for the inverse design of PLGA nanoparticles with a target size of 160 nm

Model	FRR	PLGA conc. (mg/ml)	Microchannel width (µm)
**Gradient Boosting**	0.1473	5	300
**Random Forest**	0.1500	5	300
**Polynomial (Deg 2)**	0.1267	5	300
**Ridge Regression**	0.1268	5	300
**Lasso Regression**	0.1023	5	300
**Linear Regression**	0.1271	5	300
**SVR**	0.1	5	300
**Decision Tree**	0.1500	5	300

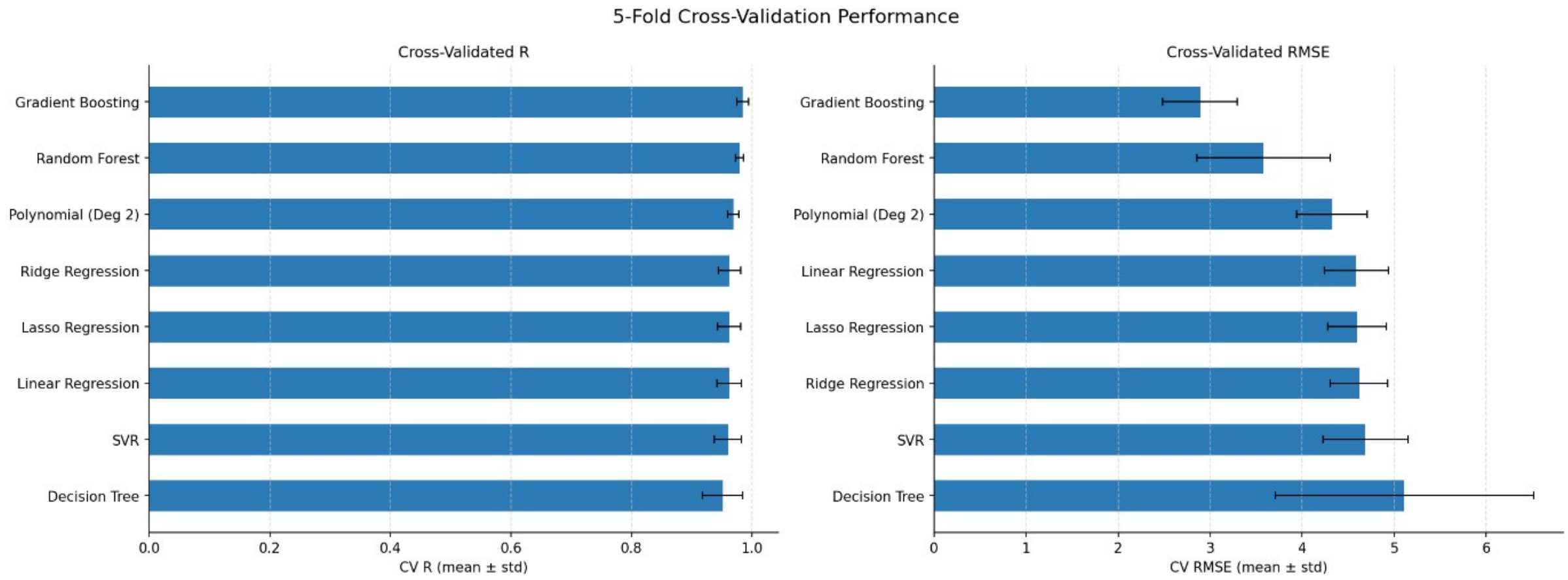

Figure S1 Five-fold cross-validation performance of the evaluated machine learning models. Comparison of the mean ± standard deviation of the cross-validated (a) coefficient of determination (R^2) and (b) root mean square error (RMSE).

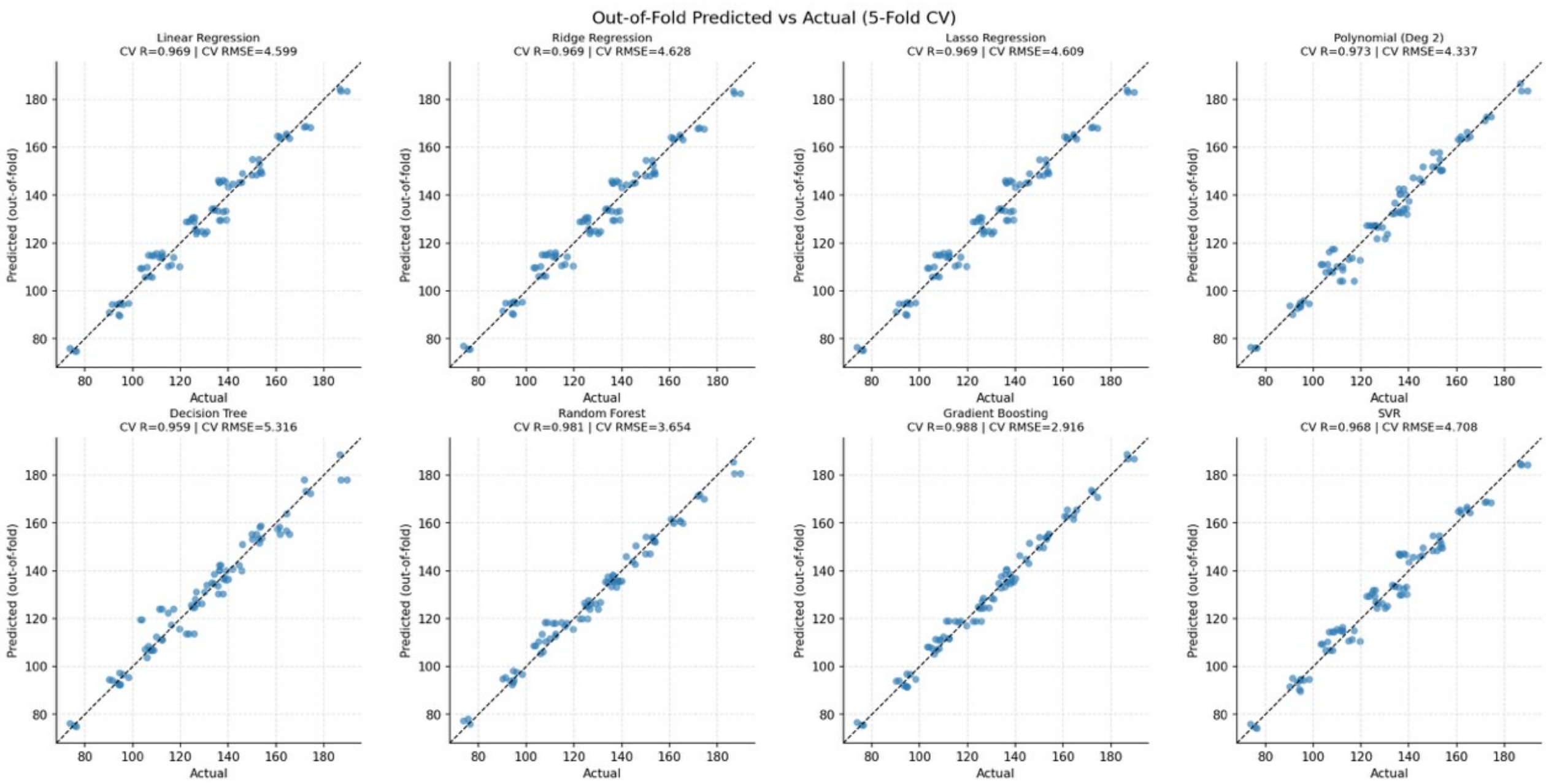

Figure S2 Out-of-fold predicted versus experimental PLGA nanoparticle sizes obtained from five-fold cross-validation for the evaluated machine learning models. The dashed line indicates the ideal 1:1 agreement between predicted and measured values.

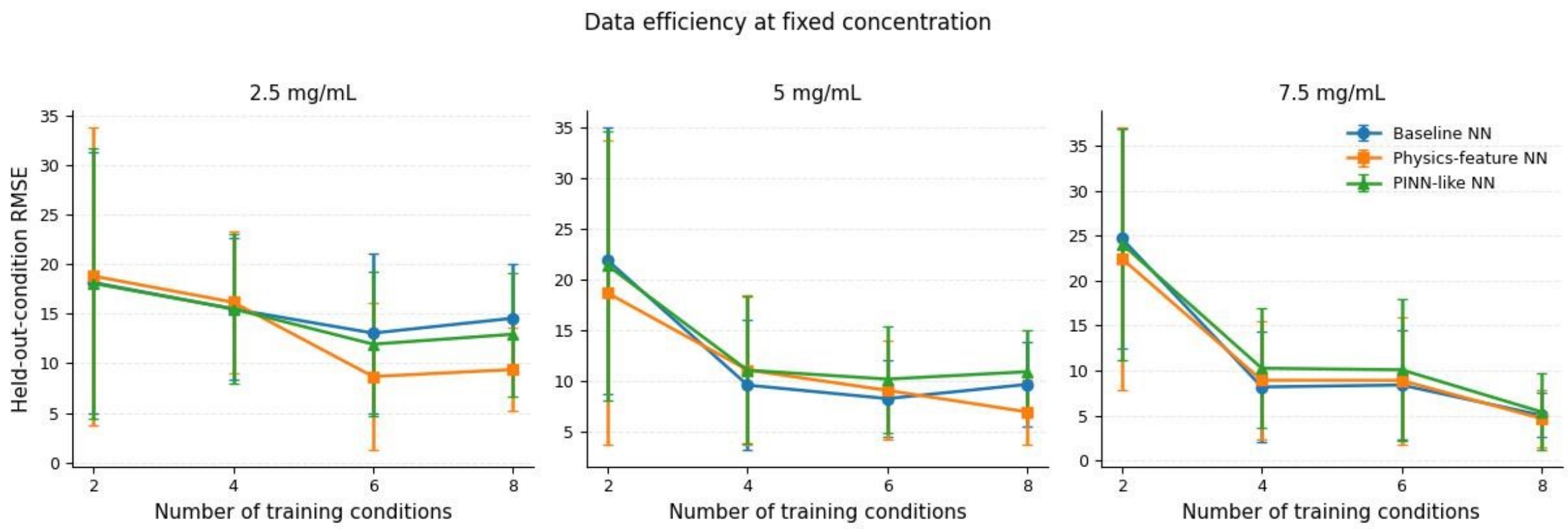

(a) (b) (c)

Figure S3 Data efficiency of neural network models at fixed concentrations. Held-out-condition RMSE as a function of the number of training conditions for the baseline NN, physics-feature NN, and PINN-like NN at 2.5, 5.0, and 7.5 mg/mL. Error bars represent the variability across held-out conditions.

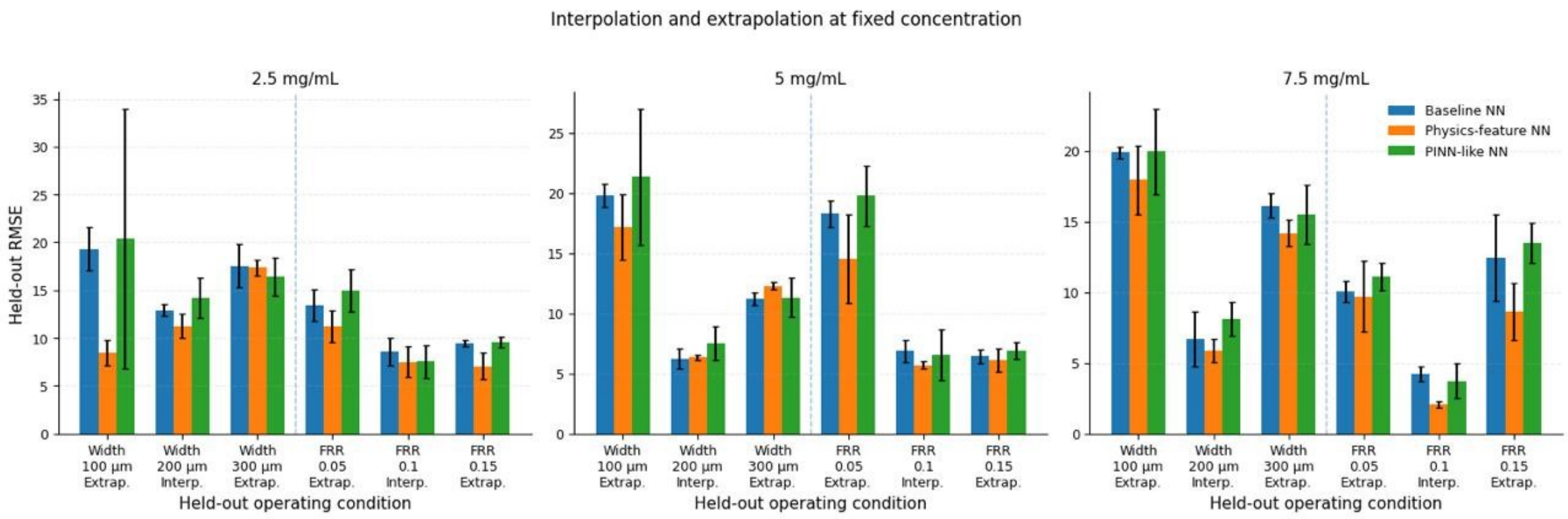

(a) (b) (c)

Figure S4 Data efficiency of neural network models at fixed concentrations. Held-out-condition RMSE as a function of the number of training conditions for the baseline NN, physics-feature NN, and PINN-like NN at 2.5, 5.0, and 7.5 mg/mL. Error bars represent the variability across held-out conditions.
